# Single and Double-Stranded 1D-Coordination Polymers with 4′-(4-Alkyloxyphenyl)-3,2′:6′,3″-terpyridines and {Cu_2_(μ-OAc)_4_} or {Cu_4_(μ_3_-OH)_2_(μ-OAc)_2_(μ_3_-OAc)_2_(AcO-κ*O*)_2_} Motifs

**DOI:** 10.3390/polym12020318

**Published:** 2020-02-04

**Authors:** Dalila Rocco, Giacomo Manfroni, Alessandro Prescimone, Y. Maximilian Klein, Dariusz J. Gawryluk, Edwin C. Constable, Catherine E. Housecroft

**Affiliations:** 1 Department of Chemistry, University of Basel, BPR 1096, Mattenstrasse 24a, CH-4058 Basel, Switzerland; dalila.rocco@unibas.ch (D.R.); giacomo.manfroni@unibas.ch (G.M.); alessandro.prescimone@unibas.ch (A.P.); edwin.constable@unibas.ch (E.C.C.); 2 Laboratory for Multiscale Materials Experiments, Paul Scherrer Institut, CH-5232 Villigen PSI, Switzerland; maximilian.klein@psi.ch (Y.M.K.); dariusz.gawryluk@psi.ch (D.J.G.)

**Keywords:** 3,2′:6′,3″-terpyridine, 1D-coordination polymer, copper(II) acetate, multinuclear cluster

## Abstract

Five coordination polymers formed from combinations of copper(II) acetate and 4′-(4-alkyloxyphenyl)-3,2′:6′,3″-terpyridines with methoxy (**1**), *n*-butoxy (**2**), *n*-pentyloxy (**3**) and *n*-heptyloxy (**4**) substituents are reported. Reaction of **1** with Cu(OAc)_2_∙H_2_O leads to the 1D-polymer [Cu_2_(μ-OAc)_4_(**1**)]*_n_* in which {Cu_2_(μ-OAc)_4_} paddle-wheel units are connected by ligands **1**, or [{Cu_4_(μ_3_-OH)_2_(μ-OAc)_2_(μ_3_-OAc)_2_(AcO-κO)_2_(**1**)_2_}·2MeOH]*_n_* in which centrosymmetric tetranuclear clusters link pairs of ligands **1** to give a double-stranded 1D-polymer. Layering solutions of Cu(OAc)_2_∙H_2_O (in MeOH) over **2**, **3** or **4** (in CHCl_3_) leads to the assembly of the 1D-polymers [2{Cu_2_(μ-OAc)_4_(**2**)}·1.25MeOH]*_n_*, [Cu_2_(μ-OAc)_4_(**3**)]*_n_* and [{Cu_2_(μ-OAc)_4_(**4**)}·0.2CHCl_3_]*_n_*. In all compounds, the 3,2′:6′,3″-tpy unit coordinates only through the outer pyridine rings, but the conformation of the 3,2′:6′,3″-tpy responds to changes in the length of the alkyloxy tails leading to changes in the conformation of the polymer backbone and in the packing of the chains in the crystal lattice in the chains featuring {Cu_2_(μ-OAc)_4_} paddle-wheel linkers.

## 1. Introduction

**Scheme 1:**
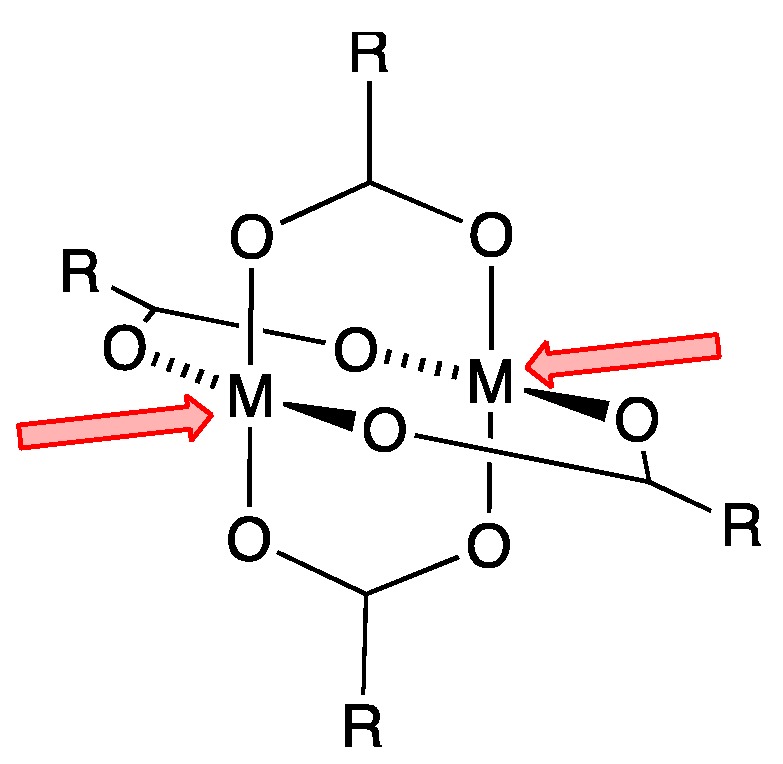
Representation of an {M_2_(μ-O_2_CR)_4_} paddle-wheel secondary building unit (SBU) with available coordination sites.

The directional assembly of coordination polymers and networks depends upon the compatibility of directing-nodes and linkers [1]. Directional metal nodes are key to the rational design of 2- and 3-dimensional inorganic materials and is at the heart of ‘reticular chemistry’ in which well-defined molecular units, so-called secondary building units (SBUs), are used to direct extended frameworks in a pre-determined and controlled fashion [2,3]. One of the simplest SBUs is the M_2_(μ-O_2_CR)_4_ paddle-wheel motif in which the axial sites of the dinuclear core provide linear directionality (Scheme 1) [4,5]. The manner in which the M_2_(μ-O_2_CR)_4_ units are connected in a coordination network depends upon the nature of the linkers, and also whether the R group in the bridging carboxylate is coordinatively innocent or not. In a simple case, where R is non-coordinating, and the organic linker is a rigid rod, such as 4,4′-bipyridine (4,4′-bpy), a topologically and structurally linear 1D-chain is assembled, as for example in [Mo_2_(μ-O_2_C^t^Bu)_4_(4,4′-bpy)]*_n_* [6]. 3D-frameworks can be achieved by retaining a rigid linker, such as 4,4′-bpy, but introducing paddle-wheel units which incorporate multifunctional carboxylate ligands as exemplified by Zou and coworkers [7]. The use of organic dicarboxylates for connected paddle-wheel motifs in combination with rigid bidentate linkers is an established strategy for MOF design [8,9]. Once we move away from rigid-rod linkers, assembly algorithms become less predictable [10].

**Scheme 2:**
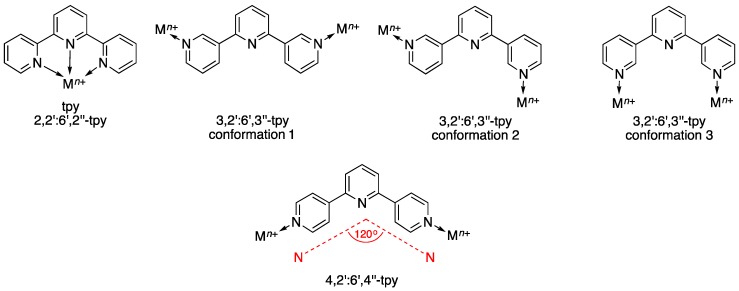
Chelating metal-binding mode of tpy (monotopic ligand) versus ditopic metal-binding modes of 3,2′:6′,3″-tpy and 4,2′:6′,4″-tpy.

We are particularly interested in the use of two isomers of terpyridine, 3,2′:6′,3″-terpyridine (3,2′:6′,3″-tpy, IUPAC PIN 1^3^,2^2^:2^6^,3^3^-terpyridine) and 4,2′:6′,4″-terpyridine (4,2′:6′,4″-tpy, PIN 1^4^,2^2^:2^6^,3^4^-terpyridine) as both organic linkers and nodes in network assemblies [11,12]. Scheme 2 illustrates that in contrast to the archetypal 2,2′:6′,2″-terpyridine (tpy, PIN 1^2^,2^2^:2^6^,3^2^-terpyridine), 3,2′:6′,3″-tpy and 4,2′:6′,4″-tpy do not bind metal ions by chelation, neither is the central nitrogen atom in 3,2′:6′,3″-tpy and 4,2′:6′,4″-tpy involved in metal coordination [13,14]. Combinations of 4,2′:6′,4″-tpy or its 4′-substituted derivatives with {M_2_(μ-O_2_CR)_4_} (M = Cu, Zn; R is non-coordinating) units leads to 1D-coordination polymers which are topologically linear, but possess a zigzag structure by virtue of the 120° angle subtended by the outer donors at the central pyridine ring (Scheme 2) [15,16,17,18,19,20]. Although paddle-wheel SBUs are typical, there is also the possibility of assembling higher nuclearity motifs. For example, the reaction of Zn(OAc)_2_·2H_2_O with 4′-(pentafluorobiphenyl-4-yl)-4,2′:6′,4″-tpy yields crystals of the expected zigzag 1D-coordination polymer [Zn_2_(μ-OAc)_4_(4′-(2′,3′,4′,5′,6′-pentafluoro-[1,1′-biphenyl]-4-yl)-4,2′:6′,4″-tpy)]*_n_* in addition to crystals of [{Zn_5_(OAc)_10_(4′-(2′,3′,4′,5′,6′-pentafluoro-[1,1′-biphenyl]-4-yl)-4,2′:6′,4″-tpy)}·11H_2_O]*_n_* in the same crystallization tube [17]. The latter comprises quadruple-stranded polymer chains supported by {Zn_5_(OAc)_10_} units. Multiple-stranded 1D-coordination polymers have also been observed when cadmium(II) acetate or manganese(II) acetate react with some 4′-substituted 4,2′:6′,4″-tpy ligands, again as a result of the assembly of non-paddle-wheel SBUs [19,21]. The presence of co-ligands often complicates the picture as in the assembly of [{Zn_6_(4-pytpy)_3_(mal)_4_}·5H_2_O]*_n_* where 4-pytpy = 4′-(4-pyridyl)-4,2′:6′,4″-terpyridine and H_2_mal = malic acid (2-hydroxybutanedioic acid) [22].

**Scheme 3:**
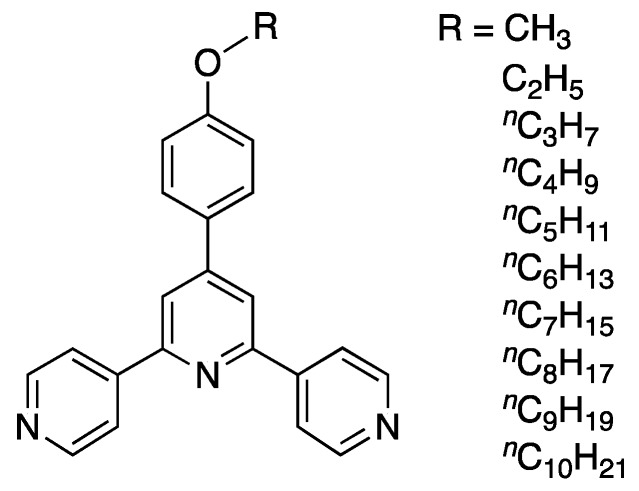
4′-(4-Alkyloxyphenyl)-4,2′:6′,4″-tpy ligands employed in a previous investigation [20].

**Scheme 4:**
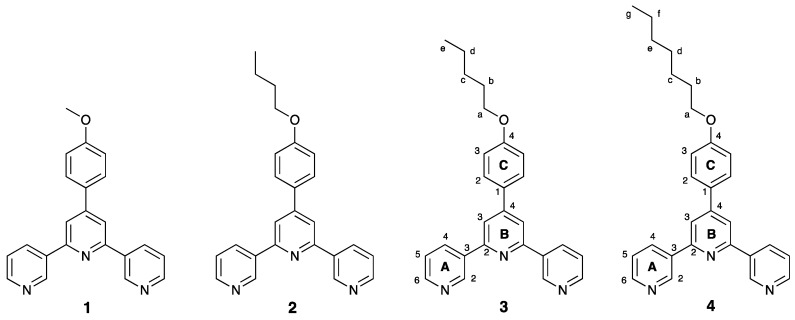
Structures of ligands **1**–**4**, with atom numbering for NMR spectroscopic assignments of **3** and **4**.

In a systematic investigation of reactions of zinc(II) acetate with the 4′-(4-alkyloxyphenyl)-4,2′:6′,4″-tpy ligands shown in Scheme 3, we observed the formation of zigzag 1D-coordination polymers [Zn_2_(μ-OAc)_4_(4′-(4-alkyloxyphenyl)-4,2′:6′,4″-tpy)]*_n_* for short chains (R = Me, Et or *^n^*Pr in Scheme 3), but for *n*-octyloxy, *n*-nonyloxy and *n*-decyloxy substituents, discrete [Zn_2_(μ-OAc)_4_(4′-(4-alkyloxyphenyl)-4,2′:6′,4″-tpy)_2_] coordination complexes were formed. For intermediate chain-lengths, the assembly pathways appear to compete with one another, and the packing of 1D-coordination polymers responds to the longer alkyloxy substituents with changes in the π-stacking motifs and by the incorporation of solvent or AcOH molecules into the crystal lattice [20]. A switch from 4,2′:6′,4″-tpy to 3,2′:6′,3″-tpy brings with it greater conformational flexibility. Both ligands possess rotational freedom about the inter-annular C–C bonds. Whereas this rotation has no effect on the vectorial arrangement of the outer-ring nitrogen lone pairs in 4,2′:6′,4″-tpy, it does cause conformational changes in 3,2′:6′,3″-tpy. The metal-binding arrangements shown in the middle diagrams in Scheme 2, are two possible vectorial arrangements of the nitrogen lone pairs. We were, therefore, interested in exploring how the structures of coordination assemblies formed from combinations of {M_2_(μ-OAc)_4_} paddle-wheel units with 4′-(4-alkyloxyphenyl)-3,2′:6′,3″-tpy ligands were influenced by changes in the length of the alkyloxy tails. Zhang and coworkers have reported that copper(II) acetate reacts with 4′-(4-*n*-hexyloxyphenyl)-3,2′:6′,3″-tpy to give a 1D-coordination polymer [23]. Coordination polymers and networks featuring ditopic 3,2′:6′,3″-tpy linkers are relatively scarce [24,25,26,27,28], although related ligands incorporating additional donors such as carboxylates and sulfonates have received some attention, as illustrated by examples in references [29,30,31]. Tetratopic ligands containing two 3,2′:6′,3″-motifs [11,32] have also been described. Herein we report the reactions of copper(II) acetate with ligands **1**–**4** (Scheme 4).

## 2. Materials and Methods

### 2.1. General

^1^H and ^13^C{^1^H} NMR spectra were recorded on a Bruker Avance iii-500 spectrometer (Bruker BioSpin AG, Fällanden, Switzerland) at 298 K. The ^1^H and ^13^C NMR chemical shifts were referenced with respect to residual solvent peaks (*δ* TMS = 0). Electrospray ionization (ESI) mass spectra were recorded using a Shimadzu LCMS-2020 instrument (Shimadzu Schweiz GmbH, Reinach, Switzerland) and samples were introduced as MeCN solutions with a drop of formic acid added. A PerkinElmer UATR Two (Perkin Elmer, Schwerzenbach, Switzerland) and Cary-5000 (Agilent Technologies Inc., Santa Clara, CA, USA) or Shimadzu UV2600 (Shimadzu Schweiz GmbH, Reinach, Switzerland) spectrophotometer were used to record FT-infrared (IR) and absorption spectra, respectively.

Compounds **1** [33] and **2** [34] were prepared as previously reported. Cu(OAc)_2_∙H_2_O was purchased from Fluka (Fluka Chemie GmbH, Buchs, Switzerland) 4-pentyloxybenzaldehyde from Combi-Blocks (Chemie Brunschwig AG, Basel, Switzerland), 4-heptyloxybenzaldehyde from Alfa Aesar (Thermo Fisher GmbH, Kandel, Germany) and were used as received.

### 2.2. Compound ***3***

4-Pentyloxybenzaldehyde (1.92 g, 1.88 mL, 10 mmol) was dissolved in EtOH (50 mL), and then 3-acetylpyridine (2.42 g, 2.20, ml 20 mmol) and crushed KOH (1.12 g, 20 mmol) were added to the solution. The color changed to orange. Aqueous NH_3_ (32%, 38.5 mL) was slowly added to the reaction mixture, and this was stirred at room temperature (ca. 22 °C) overnight. The precipitate that formed was collected by filtration, washed with EtOH (3 × 10 mL), recrystallized from EtOH and dried *in vacuo*. Compound **3** was isolated as a white powder (0.93 g, 2.23 mmol, 23.5%). M.p. = 116 °C. ^1^H NMR (500 MHz, CDCl_3_): *δ*/ppm = 9.37 (dd, *J* = 2.3, 0.9 Hz, 2H, H^A2^), 8.70 (dd, *J* = 4.8, 1.7 Hz, 2H, H^A6^), 8.51 (dt, *J* = 7.9, 2.3 Hz, 2H, H^A4^), 7.93 (s, 2H, H^B3^), 7.71 (m, 2H, H^C2^), 7.46 (ddd, *J* = 7.9, 4.8, 0.9 Hz, 2H, H^A5^), 7.07 (m, 2H, H^C3^), 4.05 (t, *J* = 6.6 Hz, 2H, H^a^), 1.84 (m, 2H, H^b^), 1.49 (m, 2H, H^c^), 1.42 (m, 2H, H^d^), 0.96 (t, *J* = 7.2 Hz 3H, H^e^). ^13^C{^1^H} NMR (500 MHz, CDCl_3_): *δ*/ppm = 160.6 (C^C4^), 155.4 (C^A3^), 150.6 (C^B4^), 150.2 (C^A6^), 148.5 (C^A2^), 135.0 (C^A4^), 134.7 (C^B2^), 130.3 (C^C1^), 128.5 (C^C2^), 123.8 (C^A5^), 117.3 (C^B3^), 115.4 (C^C3^), 68.4 (C^a^), 29.1 (C^b^), 28.4 (C^c^), 22.6 (C^d^), 14.2 (C^e^). UV-VIS (CH_3_CN, 2.5 × 10^−5^ mol dm^−3^) *λ*/nm 227 (ε/dm^3^ mol^−1^ cm^−1^ 36,500), 272 (34,400). ESI-MS *m/z* 396.20 [M+H]^+^ (calc. 396.20). Found C 78.37, H 6.34, N 10.25; required for C_26_H_25_N_3_O C 78.96, H 6.37, N 10.62.

### 2.3. Compound ***4***

4-Heptyloxybenzaldehyde (2.20 g, 2.23 mL, 10 mmol) was dissolved in EtOH (50 mL). 3-Acetylpyridine (2.42 g, 2.20 mL, 20 mmol) and crushed KOH (1.12 g, 20 mmol) were added to the solution, and then aqueous NH_3_ (32%, 38.5 mL) was slowly added. The reaction mixture was stirred at room temperature (ca. 22 °C) overnight and a precipitate formed. This was separated by filtration, washed with EtOH (3 × 10 mL), recrystallized from EtOH and dried *in vacuo*. Compound **4** was isolated as a pale yellow powder (1.22 g, 2.89 mmol, 28.9%). M.p. = 124 °C. ^1^H NMR (500 MHz, CDCl_3_): *δ*/ppm = 9.38 (dd, *J* = 2.3, 0.8 Hz, 2H, H^A2^), 8.71 (dd, *J* = 4.9, 1.7 Hz, 2H, H^A6^), 8.56 (dt, *J* = 8.0, 2.3 Hz, 2H, H^A4^), 7.94 (s, 2H, H^B3^), 7.71 (m, 2H, H^C2^), 7.50 (ddd, *J* = 8.0, 4.9, 0.8 Hz, 2H, H^A5^), 7.06 (m, 2H, H^C3^), 4.05 (t, *J* = 6.6 Hz, 2H, H^a^), 1.84 (m, 2H, H^b^), 1.49 (m, 2H, H^c^), 1.39 (m, 2H, H^d^), 1.33 (m, 4H, H^e^ + H^f^), 0.91 (m, 3H, H^g^). ^13^C{^1^H} NMR (500 MHz, CDCl_3_): *δ*/ppm = 160.7 (C^C4^), 155.1 (C^A3^), 150.7 (C^B4^), 149.5 (C^A6^), 147.9 (C^A2^), 135.3 (C^A4^), 135.2 (C^B2^), 130.1 (C^C1^), 128.5 (C^C2^), 124.0 (C^A5^), 117.5 (C^B3^), 115.4 (C^C3^), 68.4 (C^a^), 31.9 (C^e^), 29.4 (C^b^), 29.2 (C^d^), 26.2 (C^c^), 22.8 (C^f^), 14.2 (C^g^). UV-VIS (CH_3_CN, 2 × 10^−5^ mol dm^−3^) λ/nm 226 (ε/dm^3^ mol^−1^ cm^−1^ 35,400), 272 (32,500). ESI-MS *m/z* 424.26 [M+H]^+^ (calc. 424.23). Found C 79.23, H 6.85, N 9.90; required for C_28_H_29_N_3_O: C 79.40, H 6.90, N 9.92.

### 2.4. Reaction between ***1*** and Cu(OAc)_2_·H_2_O: Experiment 1

A solution of Cu(OAc)_2_∙H_2_O (12.0 mg, 0.060 mmol) in MeOH (3 mL) was layered over a CHCl_3_ solution (3 mL) of **1** (10.2 mg, 0.030 mmol) in a crystallization tube (inner diameter, i.d. = 13.6 mm, volume = 24 mL). Blue block-like crystals were obtained after two months, and one single crystal was selected for X-ray diffraction. Structural determination confirmed the formation of [Cu_2_(μ-OAc)_4_(**1**)]*_n_*. The remaining crystals in the tube were washed with MeOH and CHCl_3_, dried under vacuum and analyzed by PXRD (see the discussion section).

### 2.5. Reaction between ***1*** and Cu(OAc)_2_·H_2_O: Experiment 2

A MeOH (8 mL) solution of Cu(OAc)_2_·H_2_O (16.3 mg, 0.08 mmol) was layered over a CHCl_3_ (5 mL) solution of **1** (13.6 mg; 0.04 mmol) in a crystallization tube (i.d. = 13.6 mm, vol. = 24 mL) initially sealed with a septum; after 10 days a syringe-needle was introduced into the septum opening the tube to the air. This was left to stand at room temperature (ca. 22 °C) allowing the blue solution to evaporate in the air for two weeks. The pale blue precipitate that formed was collected by filtration, dried *in vacuo* and then redissolved in MeOH. The blue solution was left to evaporate in the air in an NMR-tube with a septum pierced with a syringe-needle. Light blue block-like crystals visible to the eye were first obtained after three months, and a single crystal was selected for X-ray diffraction after another two months. Structural determination confirmed this to be {[Cu_4_(μ_3_-OH)_2_(μ-OAc)_2_(μ_3_-OAc)_2_(AcO-κ*O*)_2_(**1**)_2_]·2MeOH}*_n_*. The remaining crystals were washed with MeOH, dried under vacuum and analyzed by PXRD confirming that the single crystal was representative of the bulk material.

### 2.6. Preparative Scale Reaction between ***1*** and Cu(OAc)_2_·H_2_O to Give [Cu_2_(OAc)_4_(***1***)]_n_

Compound **1** (70.0 mg, 0.206 mmol) was dissolved in MeOH (25 mL), then Cu(OAc)_2_·H_2_O (82.3 mg, 0.412 mmol) was added to the colorless solution. A blue solution was obtained and immediately produced a fine light blue suspension. The solid that formed was collected by filtration and then dried *in vacuo* up to constant weight (4 h). The product was isolated as a light blue powder. Yield for [Cu_2_(OAc)_4_(**1**)]*_n_* (79.3 mg, 0.11 mmol, 55%). Found C 50.94, H 4.13, N 5.75; required for C_30_H_29_N_3_O_9_: C 51.28, H 4.16, N 5.98. PXRD confirmed the product to be [Cu_2_(OAc)_4_(**1**)]*_n_* (see text).

### 2.7. Crystal Growth of [2{Cu_2_(μ-OAc)_4_(***2***)}·1.25MeOH]_n_

A solution of Cu(OAc)_2_∙H_2_O (12.0 mg, 0.060 mmol) in MeOH (8 mL) was layered over a CHCl_3_ solution (5 mL) of **2** (11.4 mg, 0.030 mmol) in a crystallization tube (i.d. = 13.6 mm, vol. = 24 mL). Green block-like crystals grew after two months, and a single crystal was selected for X-ray diffraction. Structural data confirmed a formulation of [2{Cu_2_(μ-OAc)_4_(**2**)}·1.25MeOH]*_n_*. The remaining crystals in the tube were washed with MeOH and CHCl_3_, dried under vacuum and analyzed by PXRD.

### 2.8. Crystal Growth of [Cu_2_(μ-OAc)_4_(***3***)]_n_

A solution of Cu(OAc)_2_∙H_2_O (12.0 mg, 0.060 mmol) in MeOH (3 mL) was layered over a CHCl_3_ solution (3 mL) of **3** (11.9 mg, 0.030 mmol) in a crystallization tube (i.d. = 13.6 mm, vol. = 24 mL). Blue blocks were obtained after 20 days, and a single crystal was selected for X-ray diffraction. Structural data confirmed a formulation of [Cu_2_(μ-OAc)_4_(**3**)]*_n_*. The remaining crystals in the tube were washed with MeOH and CHCl_3_, dried under vacuum and analyzed by PXRD.

### 2.9. Crystal Growth of [{Cu_2_(μ-OAc)_4_(***4***)}·0.2CHCl_3_]_n_

A solution of Cu(OAc)_2_∙H_2_O (12.0 mg, 0.060 mmol) in MeOH (6 mL) was layered over a CHCl_3_ solution (4 mL) of **4** (12.7 mg, 0.030 mmol) in a crystallization tube (i.d. = 13.6 mm, vol. = 24 mL). Blue plates grew after six months, and a single crystal was selected for X-ray diffraction. This proved to be [{Cu_2_(μ-OAc)_4_(**4**)}·0.2CHCl_3_]*_n_*. The remaining crystals were washed with MeOH and CHCl_3_, dried under vacuum and analyzed by PXRD.

### 2.10. Crystallography

Single crystal data were collected on a Bruker APEX-II diffractometer (CuKα radiation) with data reduction, solution and refinement using the programs APEX [35], ShelXT [36], Olex2 [37] and ShelXL v. 2014/7 [38], or using a STOE StadiVari diffractometer equipped with a Pilatus300K detector and with a Metaljet D2 source (GaKα radiation) and solving the structure using Superflip [39,40] and Olex2 [37]. See Section 2.10, Section 2.11, Section 2.12, Section 2.13, Section 2.14 and Section 2.15 for details of the radiation type for each structure. The model was refined with ShelXL v. 2014/7 [38]. Structure analysis used Mercury CSD v. 4.1.0 and v. 4.3.0 [41,42].

In {[Cu_4_(μ_3_-OH)_2_(μ-OAc)_2_(μ_3_-OAc)_2_(AcO-κO)_2_(**1**)_2_]·2MeOH}*_n_*, the disordered MeOH molecules have been modelled over several fractional-occupancy sites. In [{Cu_2_(μ-OAc)_4_(**4**)}·0.2CHCl_3_]*_n_*, the *n*-heptyl chain was disordered and was modelled over two sites of fractional occupancies 0.8 and 0.2; the minor occupancy site for the *n*-heptyl chain is associated with a partial occupancy CHCl_3_ molecule. The relatively high R-factor for [Cu_2_(μ-OAc)_4_(**1**)]*_n_* is due to the sharp drop in the intensity of the diffraction as a function of the resolution.

Powder X-ray diffraction (PXRD) patterns were collected at room temperature in transmission mode using a Stoe Stadi P diffractometer equipped with a Cu Kα1 radiation (Ge(111) monochromator) and a DECTRIS MYTHEN 1K detector. The reflections of the bulk samples of [{Cu_4_(μ_3_-OH)_2_(μ-OAc)_2_(μ_3_-OAc)_2_(AcO-κO)_2_(**1**)_2_}·2MeOH]*_n_* and [Cu_2_(μ-OAc)_4_(**3**)]*_n_* were indexed with the monoclinic cells *C*2/*c* and *P*2_1_/*n*, respectively. The reflections of the bulk samples of [2{Cu_2_(μ-OAc)_4_(**2**)}·1.25MeOH]*_n_* and [{Cu_2_(μ-OAc)_4_(**4**)}·0.2CHCl_3_]*_n_* and the crystalline material from the preparative scale synthesis of [Cu_2_(μ-OAc)_4_(**1**)]*_n_* were indexed with the triclinic cell *P*–1. For [{Cu_4_(μ_3_-OH)_2_(μ-OAc)_2_(μ_3_-OAc)_2_(AcO-κO)_2_(**1**)_2_}·2MeOH]*_n_* and [Cu_2_(μ-OAc)_4_(**3**)]*_n_*, Rietveld refinement analysis [43] was carried out, while for [2{Cu_2_(μ-OAc)_4_(**2**)}·1.25MeOH]*_n_*, the preparative scale synthesis of [Cu_2_(μ-OAc)_4_(**1**)]*_n_* and [{Cu_2_(μ-OAc)_4_(**4**)}·0.2CHCl_3_]*_n_*, whole-pattern decomposition (profile matching) analysis [44,45,46] of the diffraction patterns was performed with the package FULLPROF SUITE [46,47] (version July-2019) using a previously determined instrument resolution function based on a NIST640d standard. The structural models were taken from the single crystal X-ray diffraction refinements. Refined parameters in Rietveld were: Scale factor, zero shift, lattice parameters, Cu atomic positions, background points and peaks shapes as a Thompson-Cox-Hastings pseudo-Voigt function. Preferred orientations as a March-Dollase multi-axial phenomenological model were incorporated into the analysis. The refinements confirmed that the bulk materials of [{Cu_4_(μ_3_-OH)_2_(μ-OAc)_2_(μ_3_-OAc)_2_(AcO-κO)_2_(**1**)_2_}·2MeOH]*_n_* and [Cu_2_(μ-OAc)_4_(**3**)]*_n_* are representative of the analyzed single crystals.

Refined parameters in Profile matching were: Zero shift, lattice parameters, peak asymmetry, sample transparency, and peaks shapes as a Thompson-Cox-Hastings pseudo-Voigt function. The refinements confirmed that the bulk samples of [2{Cu_2_(μ-OAc)_4_(**2**)}·1.25MeOH]*_n_*, the preparative scale synthesis of [Cu_2_(μ-OAc)_4_(**1**)]*_n_* and [{Cu_2_(μ-OAc)_4_(**4**)}·0.2CHCl_3_]*_n_* are representative of the analyzed single crystals, but all show some minor impurities.

### 2.11. [Cu_2_(μ-OAc)_4_(***1***)]_n_

C_30_H_29_Cu_2_N_3_O_9_, *M*_r_ = 702.64, blue block, triclinic, space group *P*–1, *a* = 8.4817(5), *b* = 13.7510(10), *c* = 15.3776(10) Å, *α* = 115.153(5), *β* = 104.720(5), *γ* = 92.942(5)°, *V* = 1543.94(19) Å^3^, *D*_c_ = 1.511 g cm^−3^, *T* = 130 K, *Z* = 2, *Z’* = 1, μ(Ga*Kα*) = 7.754 mm^−1^. Total 23704 reflections, 6163 unique (*R_int_* = 0.1083). Refinement of 3838 reflections (402 parameters) with *I* > 2*σ*(*I*) converged at final *R*_1_ = 0.1432 (*R*_1_ all data = 0.2081), *wR*_2_ = 0.3804 (*wR*2 all data = 0.4385), gof = 1.601. CCDC 1967918.

### 2.12. [{Cu_4_(μ_3_-OH)_2_(μ-OAc)_2_(μ_3_-OAc)_2_(AcO-κO)_2_(***1***)_2_}·2MeOH]_n_

C_58_H_62_Cu_4_N_6_O_18_, *M*_r_ = 1385.29, blue block, monoclinic, space group *C*2/*c*, *a* = 26.904(2), *b* = 16.9697(15), *c* = 14.2030(13) Å, *β* = 107.436(3)°, *V* = 6186.4(10) Å^3^, *D*_c_ = 1.487 g cm^−3^, *T* = 130 K, *Z* = 4, *Z’* = 0.5, μ(CuK*α*) = 2.175 mm^−1^. Total 25907 reflections, 5690 unique (*R_int_* = 0.0369). Refinement of 5278 reflections (445 parameters) with *I* > 2*σ*(*I*) converged at final *R*_1_ = 0.0421 (*R*_1_ all data = 0.0446), *wR*_2_ = 0.1253 (*wR*2 all data = 0.1275), gof = 1.089. CCDC 1967916.

### 2.13. [2{Cu_2_(μ-OAc)_4_(***2***)}·1.25MeOH]_n_

C_67.25_H_75_Cu_4_N_6_O_19.25_, *M*_r_ = 1529.49, green block, triclinic, space group *P*–1, *a* = 14.2771(16), *b* = 16.0424(18), *c* = 17.258(2) Å, *α* = 109.126(3), *β* = 92.317(3), *γ* = 110.953(3)^o^, *V* = 3431.5(7) Å^3^, *D*_c_ = 1.480 g cm^−3^, *T* = 130 K, *Z* = 2, *Z’* = 1, μ(Cu*Kα*) = 2.032 mm^−1^. Total 61235 reflections, 12421 unique (*R_int_* = 0.0237). Refinement of 12194 reflections (886 parameters) with *I* > 2*σ*(*I*) converged at final *R*_1_ = 0.0347 (*R*_1_ all data = 0.0352), *wR*_2_ = 0.0977 (*wR*2 all data = 0.0980), gof = 1.049. CCDC 1967920.

### 2.14. [Cu_2_(μ-OAc)_4_(***3***)]_n_

C_34_H_37_Cu_2_N_3_O_9_, *M*_r_ = 758.74, blue block, monoclinic, space group *P*2_1_/*n*, *a* = 11.0414(3), *b* = 21.6148(5), *c* = 14.4787(3) Å, *β* = 106.004(2)°, *V* = 3321.53(14) Å^3^, *D*_c_ = 1.517 g cm^−3^, *T* = 130 K, *Z* = 4, *Z’* = 1, μ(Ga*Kα*) = 7.237 mm^−1^. Total 52984 reflections, 6665 unique (*R_int_* = 0.0729). Refinement of 6294 reflections (438 parameters) with *I* > 2*σ*(*I*) converged at final *R*_1_ = 0.0805 (*R*_1_ all data = 0.0867), *wR*_2_ = 0.1549 (*wR*_2_ all data = 0.1578), gof = 1.163. CCDC 1967919.

### 2.15. [{Cu_2_(μ-OAc)_4_(***4***)}·0.2CHCl_3_]_n_

C_36.2_H_41.2_Cl_0.6_Cu_2_N_3_O_9_, *M*_r_ = 810.67, blue plate, triclinic, space group *P*–1, *a* = 8.3686(6), *b* = 14.7025(11), *c* = 16.4640(13) Å, *α* = 73.217(4), *β* = 78.314(4), *γ* = 73.555(4)°, *V* = 1843.9(2) Å^3^, *D*_c_ = 1.460 g cm^−3^, *T* = 130 K, *Z* = 2, *Z’* = 1, μ(Cu*Kα*) = 2.302 mm^−1^. Total 21215 reflections, 6656 unique (*R_int_* = 0.0353). Refinement of 5906 reflections (492 parameters) with *I* > 2*σ*(*I*) converged at final *R*_1_ = 0.0514 (*R*_1_ all data = 0.0566), *wR*_2_ = 0.1495 (*wR*2 all data = 0.1553), gof = 1.048. CCDC 1967917.

## 3. Results and Discussion

### 3.1. Synthesis and Characterization of Ligands ***3*** and ***4***

**Figure 1:**
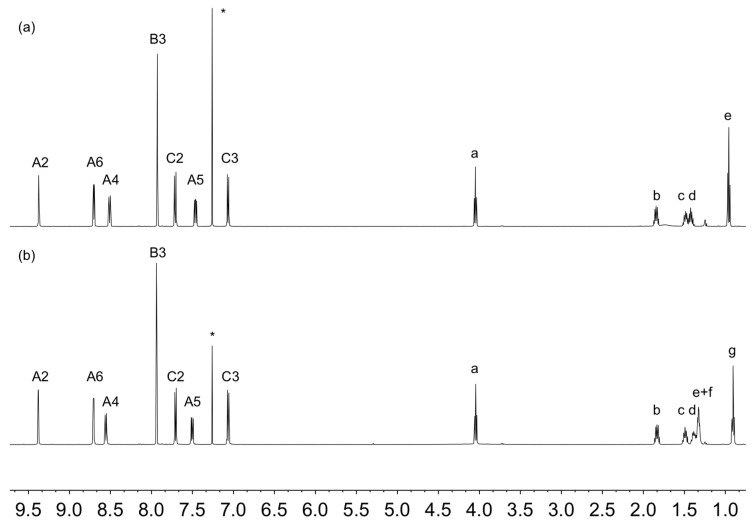
The ^1^H (500 MHz, CDCl_3_, 298 K) NMR spectra of (**a**) **3** and (**b**) **4**. * = residual CHCl_3_. See Scheme 4 for atom labelling.

Compounds **3** and **4** were synthesized using Hanan’s [48] one-pot synthesis from 3-acetylpyridine and the appropriate 4-alkyloxybenzaldehyde in the presence of base, followed by the addition of aqueous ammonia. After purification, **3** and **4** were isolated as white solids in yields of 23.5 and 28.9%, respectively. Figures S1 and S2 (see Supplementary Materials) show the electrospray mass spectra of **3** and **4**, with base peaks at *m/z* = 396.20 and 424.26, respectively, corresponding to the [M+H]^+^ ions. The solid-state IR spectra of **3** and **4** are similar (Figures S3 and S4), consistent with **3** and **4** having analogous structures. The ^1^H and ^13^C{^1^H} NMR spectra were assigned using 2D methods. Figure 1 displays a comparison of the ^1^H NMR spectra of the two compounds with the only significant difference being in the aliphatic region consistent with an *n*-pentyloxy chain in **3** versus *n*-heptyloxy chain in **4**. The HMQC and HMBC spectra are shown in Figures S5–S8.

**Figure 2:**
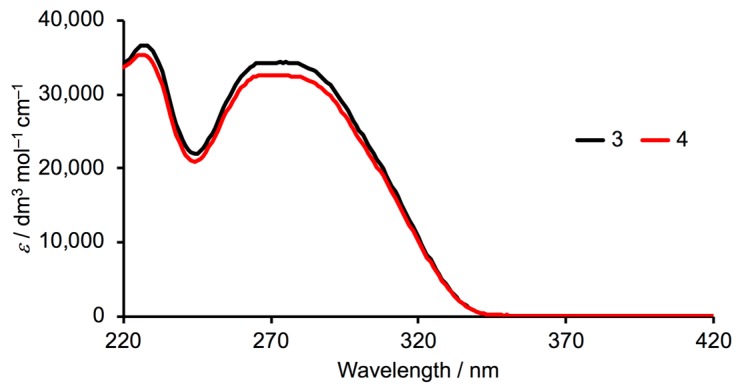
Solution absorption spectra of compounds **3** and **4** (MeCN, 2 × 10^−5^ mol dm^−3^).

The high-energy bands in the solution absorption spectra of **3** and **4** (Figure 2) arise from spin-allowed π*←*n,* and π*←π transitions, and the spectra have the same profile as those of the methoxy, ethoxy and *n*-butoxy derivatives [33,34].

### 3.2. Reactions of Copper(II) Acetate with Ligand ***1***

**Scheme 5:**
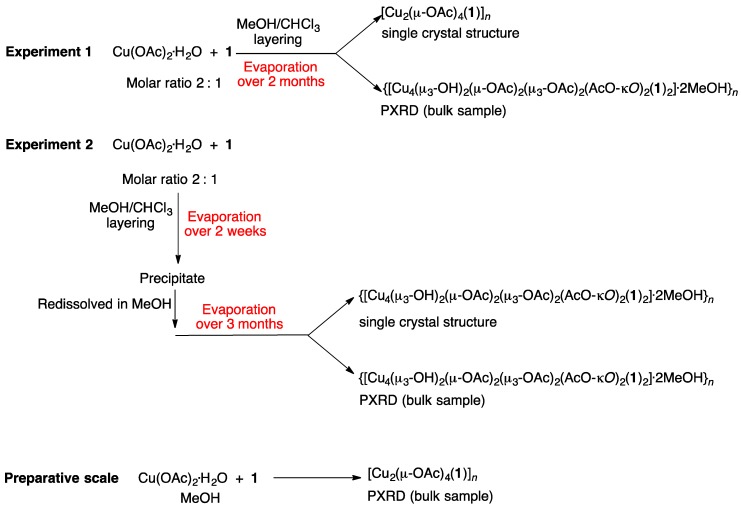
Summary of the reactions of ligand **1** with Cu(OAc)_2_∙H_2_O. Crystal growths by layering (experiments 1 and 2) and the preparative scale reactions were carried out in air at ambient temperature.

**Figure 3:**
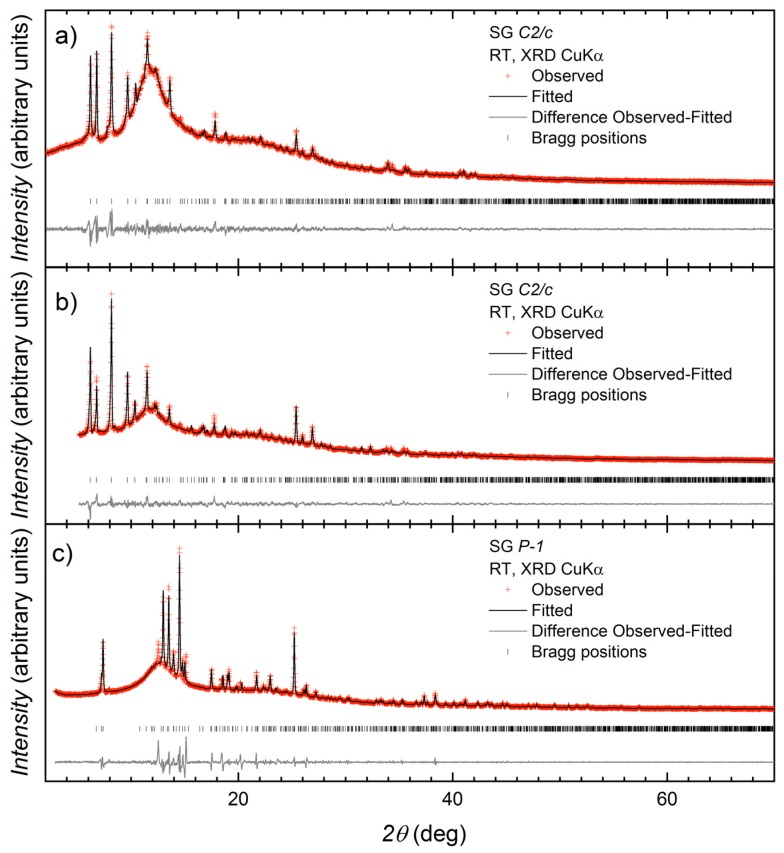
Laboratory X-ray diffraction (CuKα1 radiation) pattern (red crosses) of (**a**) the bulk crystalline material from experiment 2 with fitting to the predicted pattern from the single crystal determination of [{Cu_4_(μ_3_-OH)_2_(μ-OAc)_2_(μ_3_-OAc)_2_(AcO-κO)_2_(**1**)_2_}·2MeOH]*_n_*; (**b**) the bulk crystalline material from experiment 1 with fitting to the predicted pattern from the single crystal determination of [{Cu_4_(μ_3_-OH)_2_(μ-OAc)_2_(μ_3_-OAc)_2_(AcO-κO)_2_(**1**)_2_}·2MeOH]*_n_*; and (**c**) the crystalline material from the preparative scale synthesis with fitting to the predicted pattern from the single crystal determination of [Cu_2_(μ-OAc)_4_(**1**)]*_n_* at room temperature. The black line corresponds to the best fit from the profile matching refinement. Lower vertical marks denote the Bragg peak positions. The bottom line in each plot represents the difference between experimental and calculated points.

A series of reactions were carried out between **1** and Cu(OAc)_2_∙H_2_O, and the differences in conditions and the outcomes of the reactions are summarized in Scheme 5. All reactions were carried out in air at room temperature (ca. 22 °C). With the anticipation of the formation of dinuclear paddle-wheel units, crystallization tubes were prepared with a 2:1 molar ratio of Cu(OAc)_2_∙H_2_O:**1**, dissolved in MeOH and CHCl_3_, respectively. The tubes were left to stand at ambient temperature. Blue blocks of [Cu_2_(μ-OAc)_4_(**1**)]*_n_* suitable for single-crystal X-ray diffraction were obtained after two months from experiment 1 (see Section 2.4). In some tubes (experiment 2, Section 2.5), the solvent was allowed to evaporate more quickly by exposure to air and a precipitate formed. After filtration, the precipitate was redissolved in MeOH and the solution left to stand at room temperature. After several months, X-ray quality blue crystals were collected, and structural determination showed the formation of {[Cu_4_(μ_3_-OH)_2_(μ-OAc)_2_(μ_3_-OAc)_2_(AcO-κ*O*)_2_(**1**)_2_]·2MeOH}*_n_*. The remainder of the crystals from both Experiments 1 and 2 were collected and analyzed by PXRD. In both cases, PXRD (Figure 3a,b) confirmed that the bulk materials from the crystal growth experiments carried out over several months corresponded to {[Cu_4_(μ_3_-OH)_2_(μ-OAc)_2_(μ_3_-OAc)_2_(AcO-κ*O*)_2_(**1**)_2_]·2MeOH}*_n_*.

A preparative scale reaction of Cu(OAc)_2_·H_2_O and **1** (2:1 molar ratio) was carried out in MeOH and a pale-blue solid immediately formed. Exposure to air and moisture, in this case, was minimal. The sample was dried, and elemental analysis was in agreement with a stoichiometry of Cu(OAc)_2_(**1**), and PXRD (Figure 3c) was consistent with the material being [Cu_2_(μ-OAc)_4_(**1**)]*_n_*.

The results indicate that, on a preparative scale, ligand **1** reacts with Cu(OAc)_2_·H_2_O to immediately yield the 1D-coordination polymer [Cu_2_(μ-OAc)_4_(**1**)]*_n_*. However, when the reaction is carried out under conditions of crystal growth by layering over a period of several months, the incorporation of hydroxido ligands and formation of {Cu_4_(μ_3_-OH)_2_(OAc)_6_}-clusters becomes dominant. PXRD reveals that the double-stranded coordination polymer {[Cu_4_(μ_3_-OH)_2_(μ-OAc)_2_(μ_3_-OAc)_2_(AcO-κ*O*)_2_(**1**)_2_]·2MeOH}*_n_* is the major product in both experiments 1 and 2, and the selection of the single crystal of [Cu_2_(μ-OAc)_4_(**1**)]*_n_* was fortuitous.

**Figure 4:**
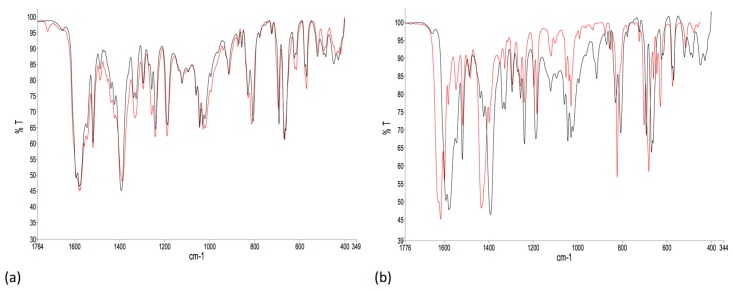
Normalized FT-IR spectra (solid-state) of the bulk materials from (**a**) experiment 1 (black line) and experiment 2 (red line), and (**b**) experiment 1 (black line, [{Cu_4_(μ_3_-OH)_2_(μ-OAc)_2_(μ_3_-OAc)_2_(AcO-κO)_2_(**1**)_2_}·2MeOH]*_n_*) and the preparative scale reaction (red line, [Cu_2_(μ-OAc)_4_(**1**)]*_n_*). Full spectra are given in Figure S9.

The IR spectra of the bulk materials from experiments 1 and 2 compared to the spectrum of the product of the preparative reaction of **1** with Cu(OAc)_2_·H_2_O are consistent with the conclusions drawn from the PXRD. Figure 4a shows an expansion of the region below 1760 cm^−1^ in the IR spectra of the bulk samples from experiments 1 and 2 confirming that the materials are the same, i.e., [{Cu_4_(μ_3_-OH)_2_(μ-OAc)_2_(μ_3_-OAc)_2_(AcO-κO)_2_(**1**)_2_}·2MeOH]*_n_*. Full spectra are shown in Figure S9 and the absorption observed at 3420 cm^−1^ is likely to arise from the O–H stretch of the hydroxido ligands [49,50]. A comparison of the IR spectra of the bulk sample from experiment 1 with that of the product from the preparative scale reaction of **1** with Cu(OAc)_2_·H_2_O is shown in Figure 4b. The absorption at 916 cm^−1^ present in the spectrum of [{Cu_4_(μ_3_-OH)_2_(μ-OAc)_2_(μ_3_-OAc)_2_(AcO-κO)_2_(**1**)_2_}·2MeOH]*_n_* (Figure 4a and black line in Figure 4b) is tentatively assigned to a vibrational mode of the μ_3_-OH group [51]. The band at 3420 cm^−1^ present in the spectrum of [{Cu_4_(μ_3_-OH)_2_(μ-OAc)_2_(μ_3_-OAc)_2_(AcO-κO)_2_(**1**)_2_}·2MeOH]*_n_* is absent in the IR spectrum of [Cu_2_(μ-OAc)_4_(**1**)]*_n_*.

### 3.3. Crystal Structure of [Cu_2_(μ-OAc)_4_(***1***)]_n_

**Figure 5:**
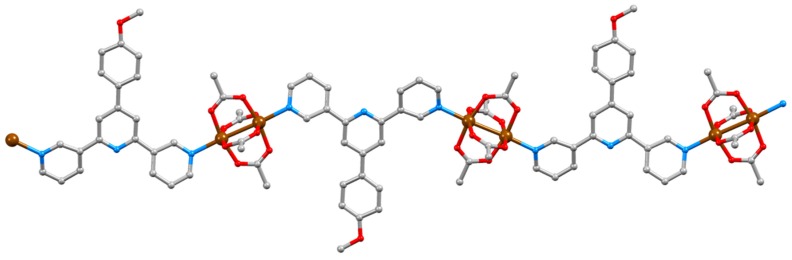
Part of one chain in [Cu_2_(μ-OAc)_4_(**1**)]*_n_*.

**Figure 6:**
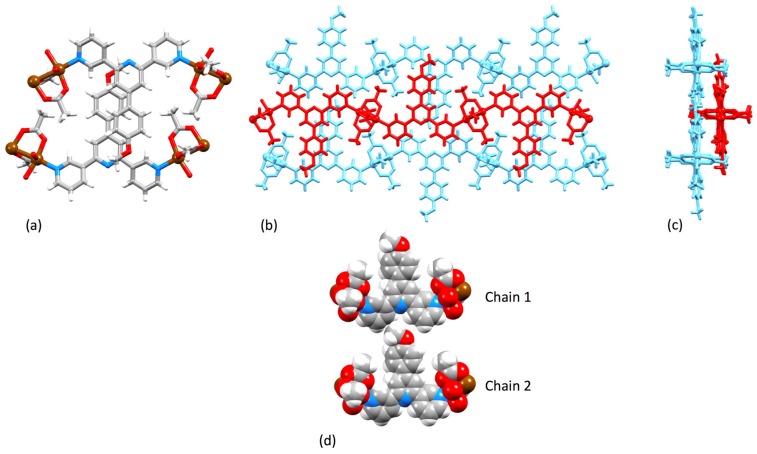
Packing in [Cu_2_(μ-OAc)_4_(**1**)]*_n_*: (**a**) Head-to-tail stacking of 4′-(4-methoxyphenyl)pyridine units in adjacent chains; (**b**) extended stacking interactions lead to the formation of 2D sheets – the red and blue chains lie in adjacent sheets; (**c**) an orthogonal view of diagram (**b**) showing the accommodation of acetato ligands in cavities in the adjacent sheet; (**d**) accommodation of a methoxy group in one chain in the pocket of a 3,2′:6′,3″-tpy domain in an adjacent chain.

[Cu_2_(μ-OAc)_4_(**1**)]*_n_* crystallizes in the triclinic space group *P*–1 and features {Cu_2_(μ-OAc)_4_} paddle-wheel units connecting ligands **1** (Figure 5). An ORTEP-style diagram of the asymmetric unit is depicted in Figure S10 (see Supplementary Materials) and Cu–N and Cu–O bond lengths are given in Table 1. Ligand **1** adopts conformation 1 in Scheme 2, resulting in a zigzag 1D chain (Figure 5). In this and the other structures described in this work, the central pyridine ring is non-coordinating. The 3,2′:6′,3″-unit is close to planar with angles between the least squares mean planes of the pyridine rings containing N1/N2 and N2/N3 being 11.1 and 17.5°, respectively. The phenyl ring is twisted only 19.5° out of the plane of the central pyridine ring. The near-planarity is a consequence of the head-to-tail stacking of the 4′-(4-methoxyphenyl)pyridine units in adjacent chains (Figure 6a). The stacked phenyl rings are offset, and although the inter-plane separation of 4.15 Å and centroid…centroid separation of 4.65 Å are larger than is ideal for face-to-face π-stacking [52], the interaction is augmented by CH_MeO_…π_pyridine_ contacts (CH…centroid = 2.91 Å). Along a 1D polymer chain, ligands **1** lie on alternate sides of the chain and extension of the stacking interactions depicted in Figure 6a leads to the formation of 2D sheets (Figure 6b). Figure 6c illustrates that two of the four acetato ligands of each {Cu_2_(μ-OAc)_4_} unit are accommodated in cavities in an adjacent sheet. An important detail with respect to the structures discussed in later sections is that the methoxy group is accommodated within the pocket of a 3,2′:6′,3″-tpy domain in the adjacent chain (Figure 6d). The closest H_MeO_…H_tpy_ distances are 2.3 and 2.8 Å. The overall efficiency of the packing in [Cu_2_(μ-OAc)_4_(**1**)]*_n_* is demonstrated by the lack of any solvent of crystallization.

**Figure 7:**
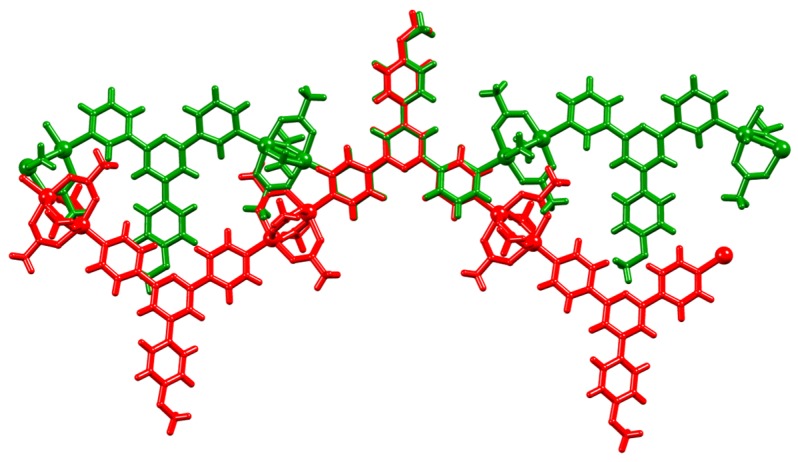
Overlay of parts of the 1D-coordination polymer chains in [Cu_2_(μ-OAc)_4_(**1**)]*_n_* (green) and [Zn_2_(μ-OAc)_4_(**1a**)]*_n_* (red). The Cambridge Structural Database, CSD [53], refcode for [Zn_2_(μ-OAc)_4_(**1a**)]*_n_* is SOXSEF.

It is pertinent to compare the 1D-coordination polymer chains in [Cu_2_(μ-OAc)_4_(**1**)]*_n_* and [Zn_2_(μ-OAc)_4_(**1a**)]*_n_* in which **1a** = 4′-(4-alkyloxyphenyl)-4,2′:6′,4″-tpy [20]. The overlay, shown in Figure 7, emphasizes the change in the vectorial arrangement of the outer pyridine-ring nitrogen lone pairs and the consequential effect on the backbone of the zigzag chain.

**Figure 8:**
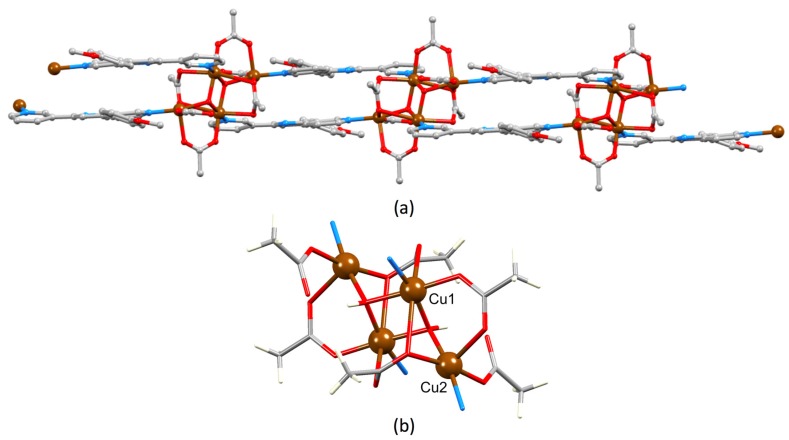
(**a**) Part of one double-stranded chain (solvent omitted) and (**b**) the cluster unit in {[Cu_4_(μ_3_-OH)_2_(μ-OAc)_2_(μ_3_-OAc)_2_(AcO-κ*O*)_2_(**1**)_2_]·2MeOH}*_n_*.

Changes to the crystallization and work-up conditions (see Materials and Method section and Section 3.2) resulted in the growth of blue crystals of {[Cu_4_(μ_3_-OH)_2_(μ-OAc)_2_(μ_3_-OAc)_2_(AcO-κ*O*)_2_(**1**)_2_]·2MeOH}*_n_*. This 1D coordination polymer crystallizes in the monoclinic space group *C*2/*c* and consists of ligands **1** (in conformation 2 in Scheme 2) linked by tetranuclear copper clusters. The asymmetric unit is depicted in Figure S11 and contains one independent ligand **1** and half of a copper cluster; the second half is generated by inversion. Consequently, the polymer is double-stranded, as shown in Figure 8a. The cluster (Figure 8b) comprises a planar array of four Cu atoms with Cu…Cu edge lengths of 3.3770(6) and 3.0822(7) Å. Two μ_3_-OH ligands (Cu–O = 1.9429(17), 1.9942(18)Å) support the rhombus (Figure 8b) and the Cu…Cu separation for the unique doubly bridged unit is 2.9863(7) Å. The hydroxido H atom was directly located (O–H = 0.841(18) Å). The edges of the Cu_4_ unit are bridged by two μ-acetato (Cu–O = 1.9406(19), 2.186(2) Å) and two μ_3_-acetato (Cu–O = 1.9879(19), 2.453(2), 2.688(2) Å) ligands; for the bridging descriptors, we consider the long Cu–O length of 2.688(2) Å to be a bonding interaction. Atom Cu2 is further coordinated by a monodentate acetato ligand (Cu–O = 1.965(2) Å). The coordination numbers of Cu1 and Cu2 are, therefore, six and five, respectively. A search of the CSD (v. 5.41 [53]) for copper clusters containing a {Cu_4_(μ_3_-OH)_2_(μ-OAc)_2_} with the same geometry as that in {[Cu_4_(μ_3_-OH)_2_(μ-OAc)_2_(μ_3_-OAc)_2_(AcO-κ*O*)_2_(**1**)_2_]·2MeOH}*_n_* revealed 75 hits, but none identical to that shown in Figure 8b. Examples of closely related clusters include those with CSD refcodes IWAHAP [54], MAFBUR [55], DUYWOL [56] and QAWHUS [57].

**Figure 9:**
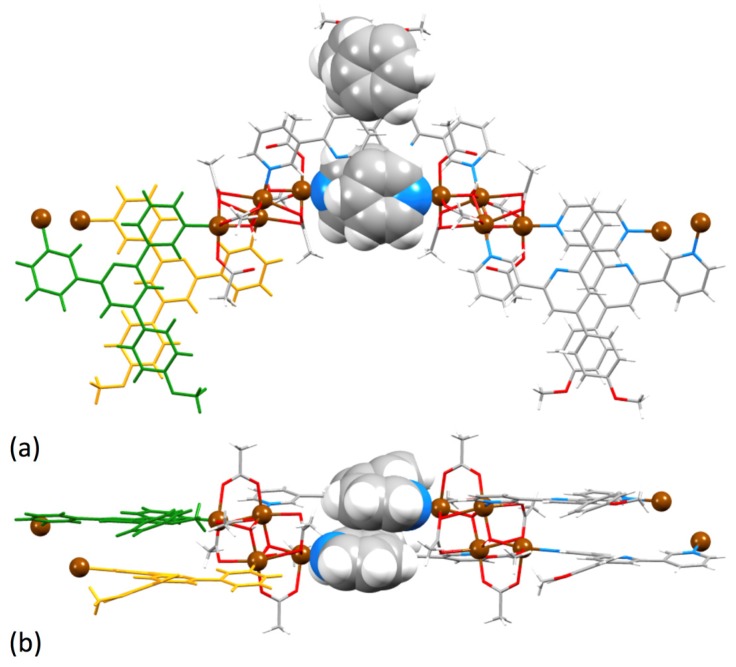
(**a**) Part of one double-stranded chain in {[Cu_4_(μ_3_-OH)_2_(μ-OAc)_2_(μ_3_-OAc)_2_(AcO-κ*O*)_2_(**1**)_2_]·2MeOH}*_n_* (solvent omitted) showing π-stacking of pairs of phenyl and pairs of pyridine rings (space-filling representation) an, in green and orange, the relative orientations of stacked ligands **1**. (**b**) The chain in diagram (a) viewed through the double-strand.

In the double-stranded chain, ligands **1** in adjacent strands lie over one another, but in an offset manner. This is illustrated in Figure 9a with green and orange coding. Pairs of 4-methoxyphenyl units engage in π-stacking with a centroid…centroid separation of 3.99 Å and an angle between the ring-planes of 8.7°. In the 3,2′:6′,3″-tpy unit, only the ring containing N2 is involved in face-to-face π-stacking; pyridine rings containing N2 and N2^i^ (symmetry code i = 1–*x*, *y*, ^3^/_2_–*z*) stack with a centroid…centroid distance of 3.69 Å and an angle between the ring-planes of 9.7° (Figure 9a,b). π-Stacking interactions also occur between polymer chains to give extended interactions through the lattice (Figure S12 in Supplementary Materials).

### 3.4. Reactions of Copper(II) Acetate with Ligands ***2***, ***3*** and ***4***

**Figure 10:**
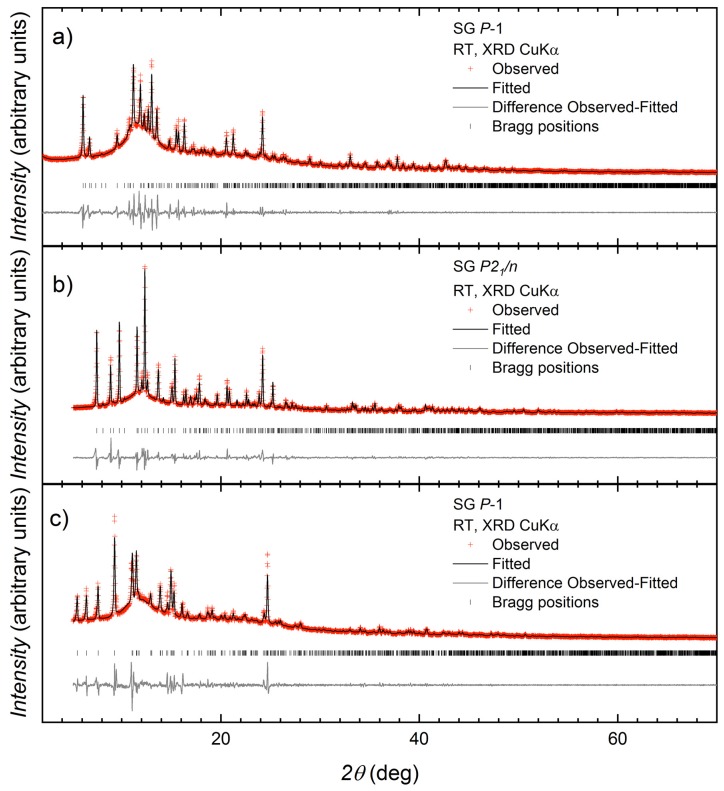
Laboratory X-ray diffraction (CuKα1 radiation) pattern (red crosses) of (**a**) [2{Cu_2_(μ-OAc)_4_(**2**)}·1.25MeOH]*_n_*_,_ (**b**) [Cu_2_(μ-OAc)_4_(**3**)]*_n_* and (c) [{Cu_2_(μ-OAc)_4_(**4**)}·0.2CHCl_3_]*_n_* at room temperature. The black line corresponds to the best fit from the profile matching refinement. Lower vertical marks denote the Bragg peak positions. The bottom line in each plot represents the difference between experimental and calculated points.

Methanol solutions of Cu(OAc)_2_∙H_2_O layered over CHCl_3_ solutions of **2**, **3** or **4** with a 2:1 molar ratio of Cu(OAc)_2_∙H_2_O:ligand yielded blue crystals. After the selection of single crystals for structure determination, the remaining crystals were analyzed by powder diffraction. PXRD refinements confirmed that the bulk material was representative of the analyzed single crystal (Figure 10a–c).

The compounds [2{Cu_2_(μ-OAc)_4_(**2**)}·1.25MeOH]*_n_* and [{Cu_2_(μ-OAc)_4_(**4**)}·0.2CHCl_3_]*_n_* crystallize in the triclinic space group *P*–1, while [Cu_2_(μ-OAc)_4_(**3**)]*_n_* crystallizes in the monoclinic space group *P*2_1_/*n*. All three compounds are 1D coordination polymers with the 4′-substituted 3,2′:6′,3″-tpy ligands linking {Cu_2_(μ-OAc)_4_} paddle-wheel units. The asymmetric units of the structures are shown in Figures S13–S15. In [2{Cu_2_(μ-OAc)_4_(**2**)}·1.25MeOH]*_n_*, there are two independent ligands and two independent {Cu_2_(μ-OAc)_4_} units. In the latter, the Cu–O bond lengths lie in the range 1.9544(15)–1.9971(15) Å (Table 1) and the geometries of the paddle-wheel units are similar. The crystallographically independent ligands **2** differ in the conformations of the *n*-butyl chains, only one is fully extended. Cu–O and Cu–N bond lengths for all three structures are given in Table 1 and are unexceptional. The alkyloxy chains in [{Cu_2_(μ-OAc)_4_(**4**)}·0.2CHCl_3_]*_n_* and [Cu_2_(μ-OAc)_4_(**3**)]*_n_* are in partly extended conformations.

**Scheme 6:**
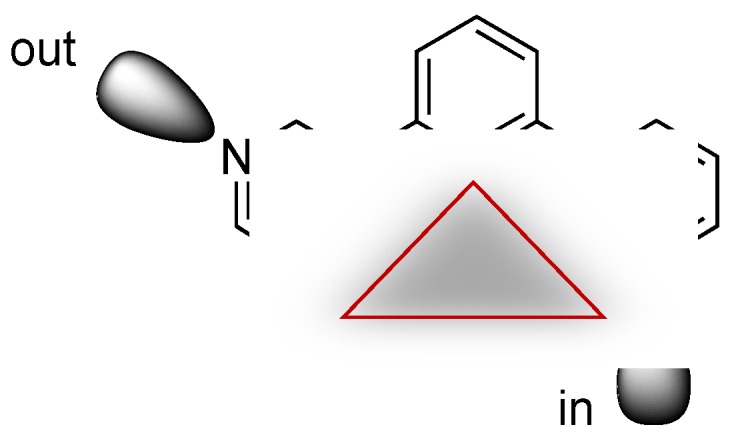
With 3,2′:6′,3″-tpy in conformation 2 (defined in Scheme 2), the outer nitrogen lone pairs point in or out with respect to the cavity (red triangle) defined by the central pyridine ring.

**Figure 11:**
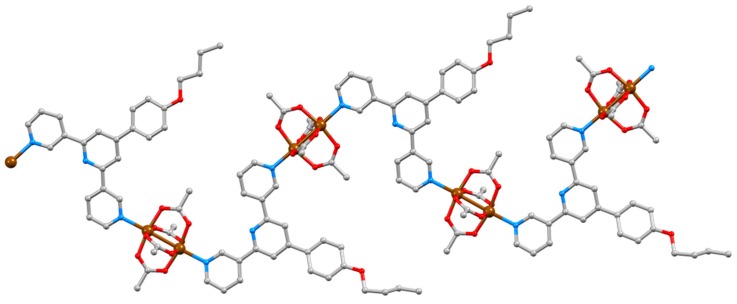
Part of one chain in [2{Cu_2_(μ-OAc)_4_(**2**)}·1.25MeOH]*_n_* with a coordination sequence of *out/in* at each paddle-wheel unit; H atoms and solvent molecules omitted for clarity.

**Figure 12:**
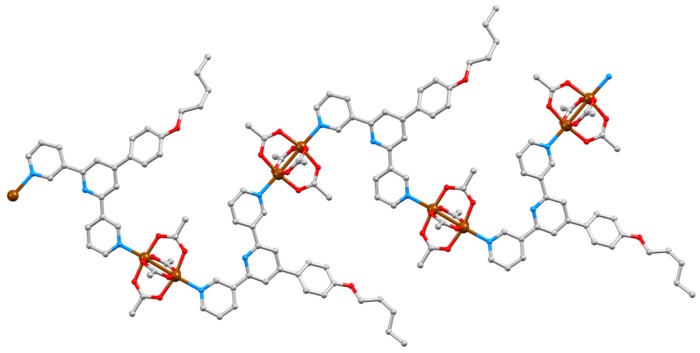
Part of one chain in [Cu_2_(μ-OAc)_4_(**3**)]*_n_* with a coordination sequence of *out/in* at each paddle-wheel unit; H atoms are omitted.

**Figure 13:**
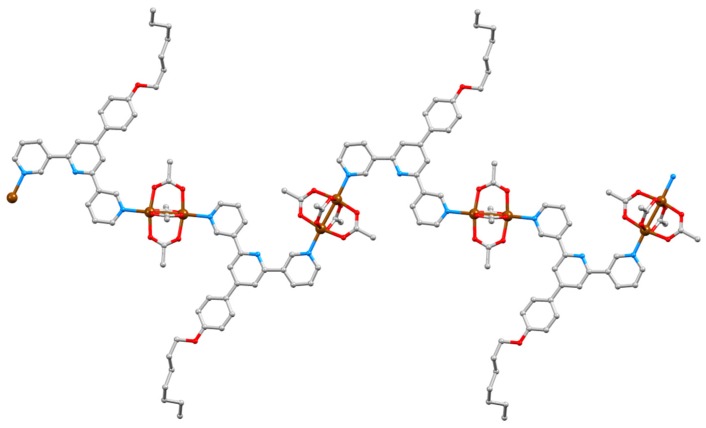
Part of one chain in [{Cu_2_(μ-OAc)_4_(**4**)}·0.2CHCl_3_]*_n_* with a coordination sequence of alternating *out/out* and *in/in* at each paddle-wheel unit. The *n*-heptyl chain is disordered. and only the major occupancy site is shown; H atoms and solvent molecules omitted for clarity.

The 1D coordination polymer chains in [2{Cu_2_(μ-OAc)_4_(**2**)}·1.25MeOH]*_n_*, [{Cu_2_(μ-OAc)_4_(**4**)}·0.2CHCl_3_]*_n_* and [Cu_2_(μ-OAc)_4_(**3**)]*_n_* are displayed in Figure 11, Figure 12 and Figure 13, respectively. In each, the 3,2′:6′,3″-tpy domain exhibits conformation 2 (Scheme 2) in contrast to conformation 1 (Scheme 2) observed in [Cu_2_(μ-OAc)_4_(**1**)]*_n_*. However, a comparison of Figure 11, Figure 12 and Figure 13 reveals a further distinction between the coordination modes. In ligand-conformation 2, the outer nitrogen lone pairs point *in* or *out* with respect to the cavity defined by the central pyridine ring of the 3,2′:6′,3″-tpy unit (Scheme 6). If we consider the axial coordination sites of the paddle-wheel units in Figure 11 (working left to right across the diagram), we can define a coordination pattern along the chain as *out/in* for each {Cu_2_(μ-OAc)_4_} unit in [2{Cu_2_(μ-OAc)_4_(**2**)}·1.25MeOH]*_n_*. Similarly, in Figure 12, the coordination sequence is *out/in* for each {Cu_2_(μ-OAc)_4_} unit in [Cu_2_(μ-OAc)_4_(**3**)]*_n_*. However, in Figure 13, the chain in [{Cu_2_(μ-OAc)_4_(**4**)}·0.2CHCl_3_]*_n_* has the sequence *out/out* for one {Cu_2_(μ-OAc)_4_} unit followed by *in/in* for the next, and so on. The structure of the missing member of this series [{Cu_2_(μ-OAc)_4_(**5**)}·0.5MeOH]*_n_* where **5** = 4′-(4-*n*-hexyloxyphenyl)-3,2′:6′,3″-tpy, has been reported by Zhang and coworkers [23]. As Figure S16 illustrates (see Supplementary Materials), the polymer chain possesses the same *in/in/out/out*... arrangement as we observe in the *n*-heptyloxy analogue [{Cu_2_(μ-OAc)_4_(**4**)}·0.2CHCl_3_]*_n_*. The packing discussion that follows helps to rationalize the dependence of the coordination mode on the length of the alkyloxy tail.

**Figure 14:**
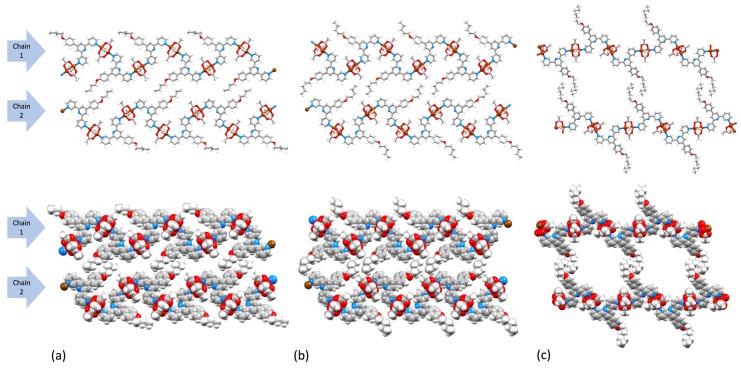
Packing of two adjacent chains (each runs left to right), shown in ball-and-stick and space-filling representations in (**a**) [2{Cu_2_(μ-OAc)_4_(**2**)}·1.25MeOH]*_n_*, (**b**) [Cu_2_(μ-OAc)_4_(**3**)]*_n_*, and (**c**) [{Cu_2_(μ-OAc)_4_(**4**)}·0.2CHCl_3_]*_n_*. Solvent molecules in (**a**) and (**c**) are omitted. In (**c**), the *n*-heptyl chain is disordered, and only the major occupancy site is shown.

In each of the three structures, the 1D coordination polymer chains pack side-by-side to form 2D sheets. Figure 14 compares the packing of two adjacent chains (each runs left to right in the figure as defined by the blue arrows) in [2{Cu_2_(μ-OAc)_4_(**2**)}·1.25MeOH]*_n_* (Figure 14a), [Cu_2_(μ-OAc)_4_(**3**)]*_n_* (Figure 14b) and [{Cu_2_(μ-OAc)_4_(**4**)}·0.2CHCl_3_]*_n_* (Figure 14c). At first glance, Figure 14a,b indicate similar packing, but closer inspection reveals a translational shift of chain 2 with respect to chain 1 on going from Figure 14a,b. While the *n*-butoxy chains are aligned to optimize van der Waals packing interactions (Figure 14a and Figure S17), pairs of *n*-pentyloxy chains are offset, and a potential interaction is partially interrupted by an acetato methyl group (Figure 14b). The space-filling representation in Figure 14b shows a centrosymmetric embrace between *n*-pentyloxy and methyl units. With an increase in the length of the alkyloxy tail to *n*-heptyloxy, the packing of polymer chains in a 2D sheet undergoes a significant change (Figure 14c) with van der Waals interactions between partly extended *n*-heptyloxy tails being the dominant in-sheet interactions. The cavity visible in Figure 14c is occupied, not by solvent molecules, but by ligands **4** from the next sheet.

**Figure 15:**
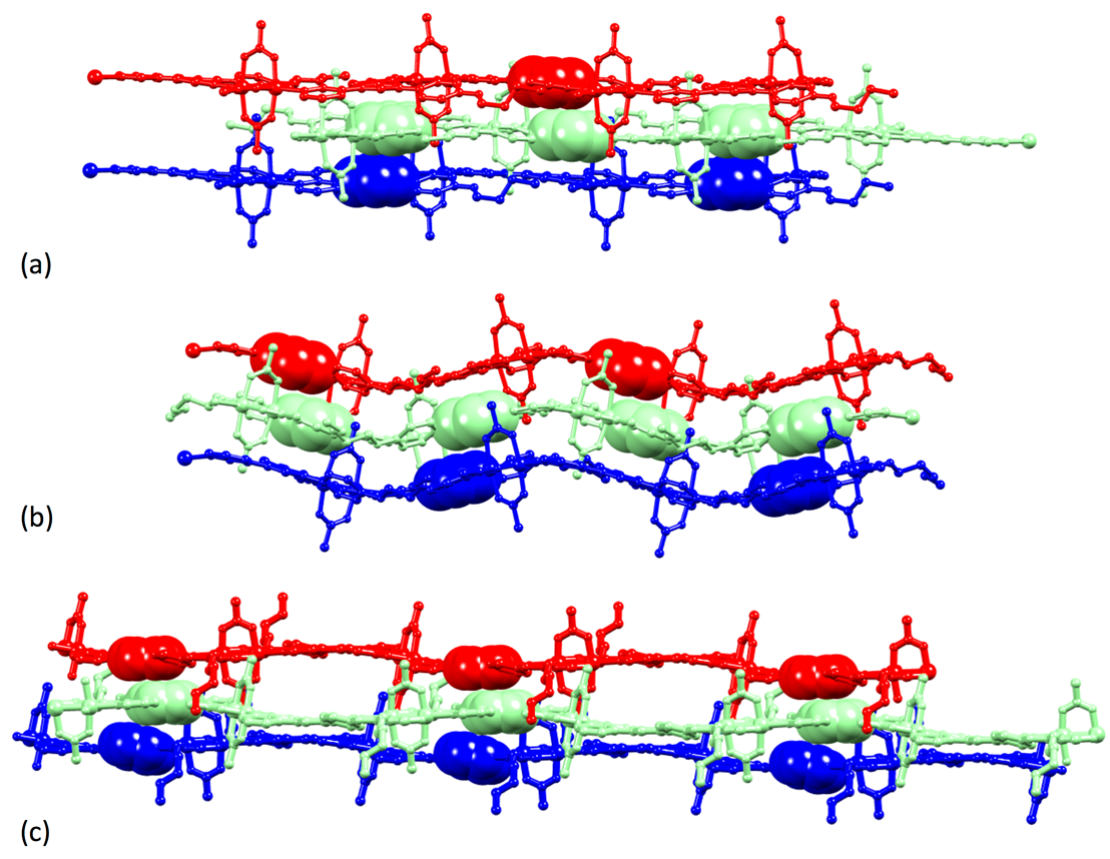
Packing of three adjacent 2D sheets (each comprising 1D polymer chains) in (**a**) [2{Cu_2_(μ-OAc)_4_(**2**)}·1.25MeOH]*_n_*, (**b**) [Cu_2_(μ-OAc)_4_(**3**)]*_n_* and (**c**) [{Cu_2_(μ-OAc)_4_(**4**)}·0.2CHCl_3_]*_n_*_._ Solvent molecules and H atoms are omitted.

The differences in inter-sheet packing in [2{Cu_2_(μ-OAc)_4_(**2**)}·1.25MeOH]*_n_*, [Cu_2_(μ-OAc)_4_(**3**)]*_n_* and [{Cu_2_(μ-OAc)_4_(**4**)}·0.2CHCl_3_]*_n_* are illustrated in Figure 15. In [2{Cu_2_(μ-OAc)_4_(**2**)}·1.25MeOH]*_n_*, each sheet comprising side-by-side packed 1D-polymer chains is essentially planar, and pairs of adjacent sheets interact through face-to-face π-stacking between centrosymmetric pairs of central pyridine rings of ligands **2** (inter-plane separation = 3.30 Å, centroid…centroid distance = 3.53 Å) (Figure 15a). The packing is similar in [Cu_2_(μ-OAc)_4_(**3**)]*_n_* (Figure 15b) with an inter-plane separation between the π-stacked pyridine rings of 3.63 Å and a centroid…centroid separation of 4.33 Å. However, Figure 15b shows that the sheets in [Cu_2_(μ-OAc)_4_(**3**)]*_n_* are ruffled in contrast to the planar sheets in [2{Cu_2_(μ-OAc)_4_(**2**)}·1.25MeOH]*_n_*. The packing undergoes a more significant change on going to [{Cu_2_(μ-OAc)_4_(**4**)}·0.2CHCl_3_]*_n_* (Figure 15c). Face-to-face interactions involve phenyl…pyridine…pyridine π-stacking.

## 4. Conclusions

We have investigated the reactions of the 4′-(4-alkyloxyphenyl)-3,2′:6′,3″-terpyridines **1**–**4** with Cu(OAc)_2_∙H_2_O. With a methoxy substituent (ligand **1**), we observe both the formation of [Cu_2_(μ-OAc)_4_(**1**)]*_n_* (which contains the ubiquitous {Cu_2_(μ-OAc)_4_} paddle-wheel motifs) and [{Cu_4_(μ_3_-OH)_2_(μ-OAc)_2_(μ_3_-OAc)_2_(AcO-κO)_2_(**1**)_2_}·2MeOH]*_n_* in which centrosymmetric tetranuclear clusters link pairs of ligands **1** to give a double-stranded 1D-polymer. PXRD confirmed that a preparative scale reaction of **1** with Cu(OAc)_2_∙H_2_O (1:2 molar ratio) immediately yields the blue microcrystalline 1D-coordination polymer [Cu_2_(μ-OAc)_4_(**1**)]*_n_*. However, crystal-growth conditions carried out by layering under ambient conditions in air over a period of several months yield predominantly the double-stranded 1D-coordination polymer [{Cu_4_(μ_3_-OH)_2_(μ-OAc)_2_(μ_3_-OAc)_2_(AcO-κO)_2_(**1**)_2_}·2MeOH]*_n_*, with [Cu_2_(μ-OAc)_4_(**1**)]*_n_* as a minor product. Crystal growth by layering a MeOH solution of Cu(OAc)_2_∙H_2_O over a CHCl_3_ solution of **2**, **3** or **4** produces blue crystals of [2{Cu_2_(μ-OAc)_4_(**2**)}·1.25MeOH]*_n_*, [Cu_2_(μ-OAc)_4_(**3**)]*_n_* or [{Cu_2_(μ-OAc)_4_(**4**)}·0.2CHCl_3_]*_n_*, all of which are single-stranded 1D coordination polymers.

In all five compounds, only the outer pyridine rings of the 3,2′:6′,3″-tpy unit coordinate to copper(II). However, in the polymers containing the {Cu_2_(μ-OAc)_4_} paddle-wheel units, the conformation of the 3,2′:6′,3″-tpy unit responds to changes in the length of the alkyloxy tails. In [Cu_2_(μ-OAc)_4_(**1**)]*_n_*, **1** adopts conformation 1 in Scheme 2, and a zigzag 1D chain results. In the polymers containing ligands **2**, **3** and **4**, the 3,2′:6′,3″-tpy adopts conformation 2 in Scheme 2, and van der Waals packing forces between alkyloxy chains become important, complementing the π-stacking interactions between phenyl… pyridine and pyridine…pyridine rings.

## Figures and Tables

**Table 1:** Selected bond lengths in the {Cu_2_(μ-OAc)_4_}-containing coordination polymers.

Coordination Polymer	Cu–O/Å	Cu–N/Å
[Cu_2_(μ-OAc)_4_(**1**)]*_n_*	1.964(8), 1.977(8), 1.987(9), 1.987(9), 1.943(8), 1.984(9), 1.953(9), 1.965(9)	2.150(9), 2.156(8)
[2{Cu_2_(μ-OAc)_4_(**2**)}·1.25MeOH]*_n_*	1.9659(16), 1.9764(16), 1.9728(15), 1.9777(16), 1.9786(15), 1.9803(15), 1.9753(15), 1.9544(15), 1.9805(16), 1.9671(15), 1.9971(15), 1.9580(15), 1.9745(16), 1.9808(15), 1.9716(16), 1.9839(15)	2.1672(18), 2.1717(17), 2.1875(17), 2.1709(17)
[Cu_2_(μ-OAc)_4_(**3**)]*_n_*	1.964(4), 1.992(4), 1.959(4), 1.988(4), 1.951(4), 1.950(4), 1.992(4), 1.997(4)	2.205(4), 2.161(4)
[{Cu_2_(μ-OAc)_4_(**4**)}·0.2CHCl_3_]*_n_*	1.963(2), 1.967(2), 1.976(2), 1.976(2), 1.976(2), 1.974(2), 1.982(2), 1.972(2)	2.163(3), 2.149(3)

## Supplementary Materials

The following are available online at https://www.mdpi.com/2073-4360/12/2/318/s1. Figures S1 and S2: Mass spectra of **3** and **4**; Figures S3 and S4: Solid-state IR spectra of **3** and **4**; Figures S5–S8: HMQC and HMBC spectra of **3** and **4**; Figure S9: FTIR spectra; Figures S10 and S11: ORTEP diagrams of the asymmetric units in [Cu_2_(μ-OAc)_4_(**1**)]*_n_* and {[Cu_4_(μ_3_-OH)_2_(μ-OAc)_2_(μ_3_-OAc)_2_(AcO-κ*O*)_2_(**1**)_2_]·2MeOH}*_n_*; Figure S12: Packing of chains in {[Cu_4_(μ_3_-OH)_2_(μ-OAc)_2_(μ_3_-OAc)_2_(AcO-κ*O*)_2_(**1**)_2_]·2MeOH}*_n_*; Figures S13–S15: ORTEP diagrams of the asymmetric units in [2{Cu_2_(μ-OAc)_4_(**2**)}·1.25MeOH]*_n_*, [Cu_2_(μ-OAc)_4_(**3**)]*_n_* and [Cu_2_(μ-OAc)_4_(**4**)}·0.2CHCl_3_]*_n_*; Figure S16: Part of one chain in [Cu_2_(μ-OAc)_4_(**5**)}·0.2CHCl_3_]*_n_*; Figure S17: Packing of chains in [2{Cu_2_(μ-OAc)_4_(**2**)}·1.25MeOH]*_n_*.
