# Supplementary material for: Single and Double-Stranded 1D-Coordination Polymers with 4′-(4-Alkyloxyphenyl)-3,2′:6′,3″-terpyridines and {Cu2(μ-OAc)4} or {Cu4(μ3-OH)2(μ-OAc)2(μ3-OAc)2(AcO-κO)2} Motifs
Source: Polymers (Basel). 2020 Feb 4;12(2):318. doi: 10.3390/polym12020318 (PMC7077432; doi:10.3390/polym12020318)
Supplement: Supplementary file 1 [file polymers-12-00318-s001.pdf]

Supporting Information

Single and double-stranded 1D-coordination polymers with 4'-(4-alkyloxyphenyl)-3,2':6',3''-terpyridines and {$Cu_2(\mu$-$OAc)_4$} or {$Cu_4(\mu_3$-$OH)_2(\mu$-$OAc)_2(\mu_3$-$OAc)_2(AcO$-$\kappa O)_2$} motifs

Dalila Rocco [1], Giacomo Manfroni [1], Alessandro Prescimone [1], Y. Maximilian Klein[2], Dariusz J. Gawryluk[2], Edwin C. Constable [1] and Catherine E. Housecroft[1]*

[1]Department of Chemistry, University of Basel, BPR 1096, Mattenstrasse 24a, CH-4058 Basel, Switzerland

[2]Laboratory for Multiscale Materials Experiments, Paul Scherrer Institut, CH-5232 Villigen PSI, Switzerland

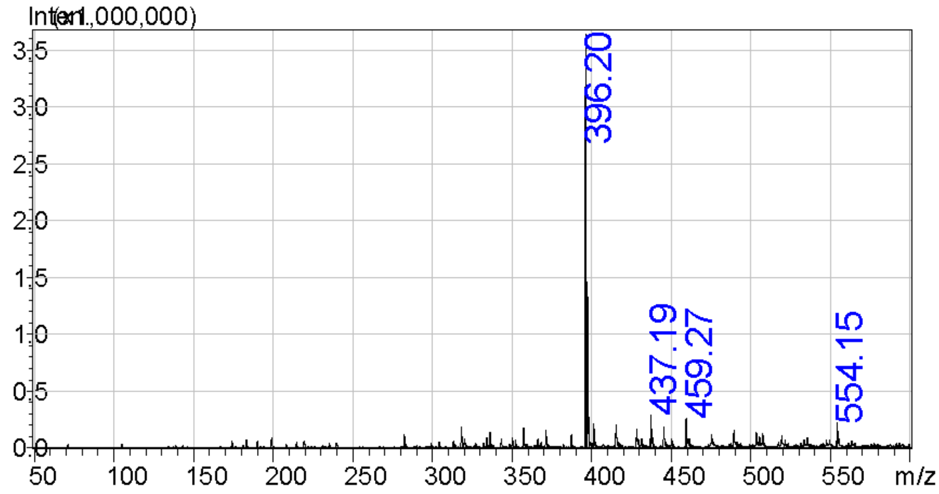

Figure S1. Electrospray mass spectrum of compound **3**.

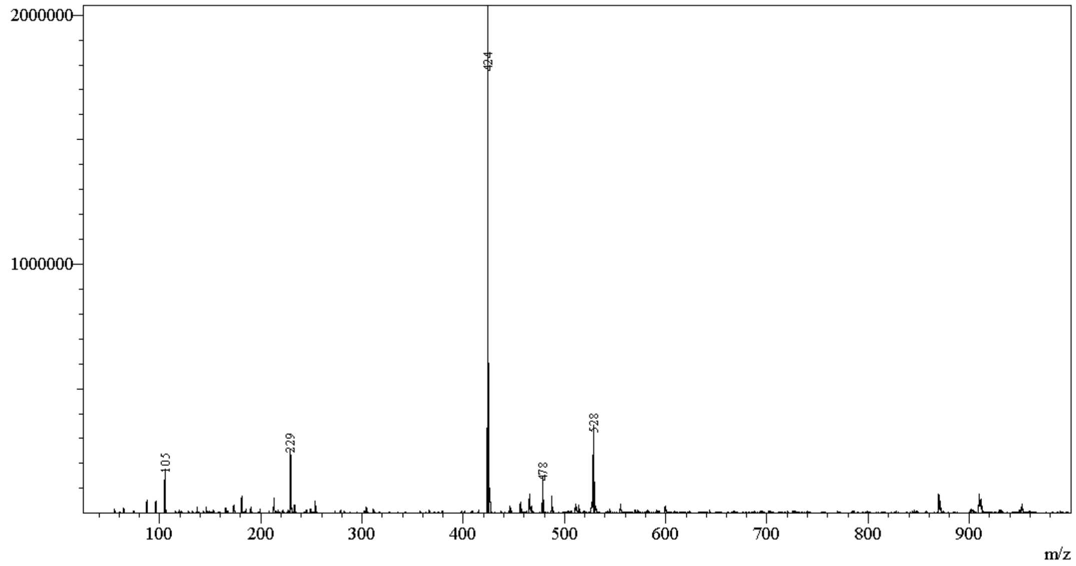

Figure S2. Electrospray mass spectrum of compound **4**.

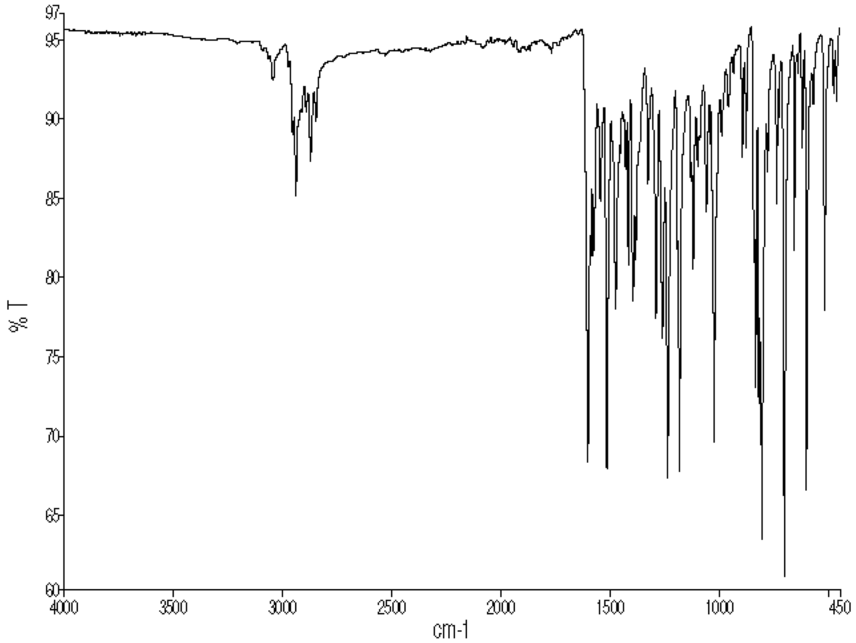

Figure S3. FT-IR spectrum of compound **3** (solid state).

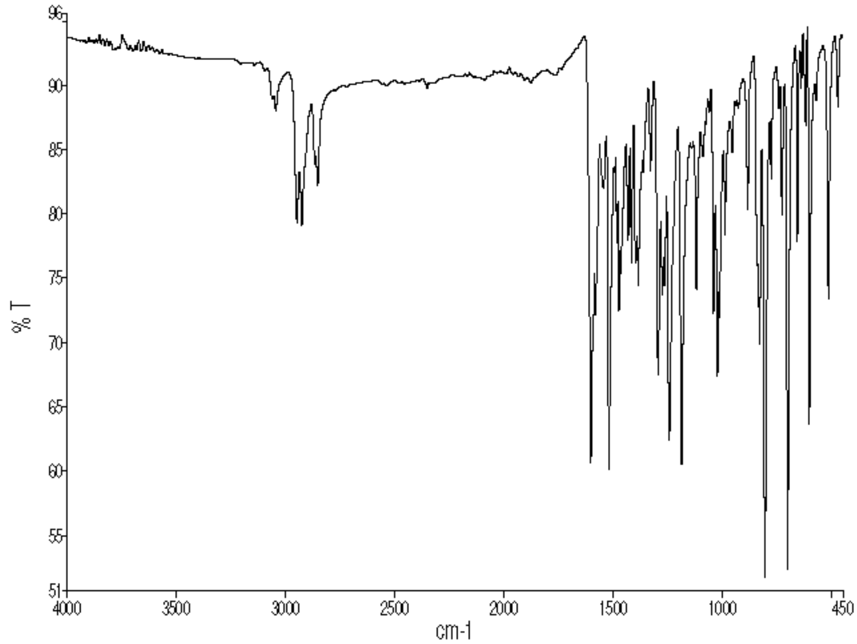

Figure S4. FT-IR spectrum of compound **4** (solid state).

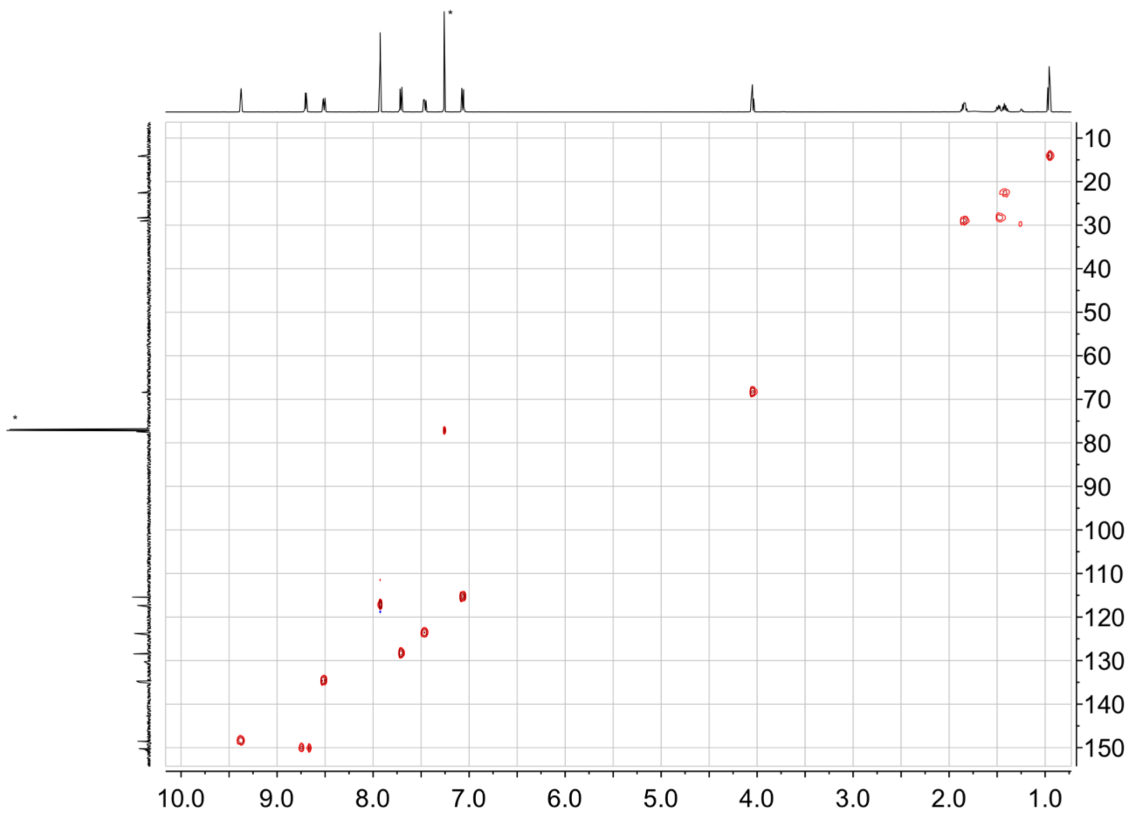

Figure S5. HMQC spectrum of **3** in $CDCl_3$ (500 MHz ^1H, 126 MHz $^{13}C\{^1H\}$, 298 K). * = $CHCl_3/CDCl_3$.

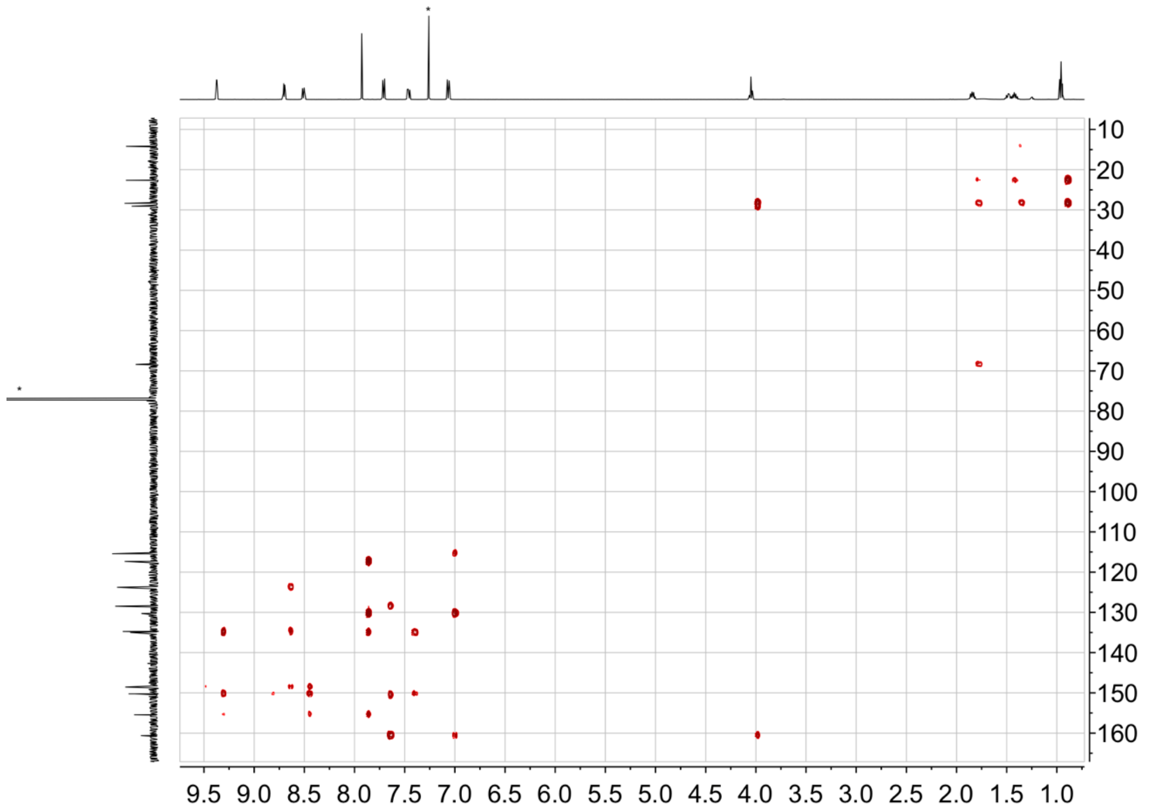

Figure S6. HMBC spectrum of **3** in $CDCl_3$ (500 MHz ^1H, 126 MHz $^{13}C\{^1H\}$, 298 K). * = $CHCl_3/CDCl_3$.

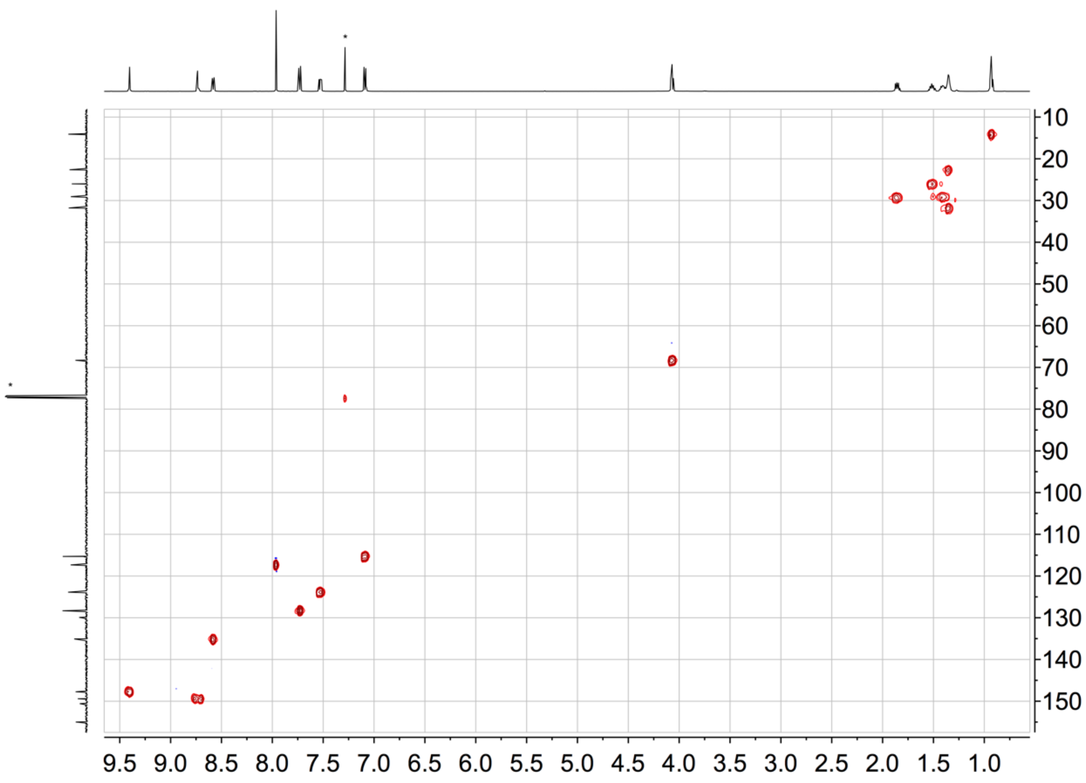

Figure S7. HMQC spectrum of **4** in $CDCl_3$ (500 MHz ^{1}H, 126 MHz ^{13}C{^{1}H}, 298 K). * = $CHCl_3$/$CDCl_3$.

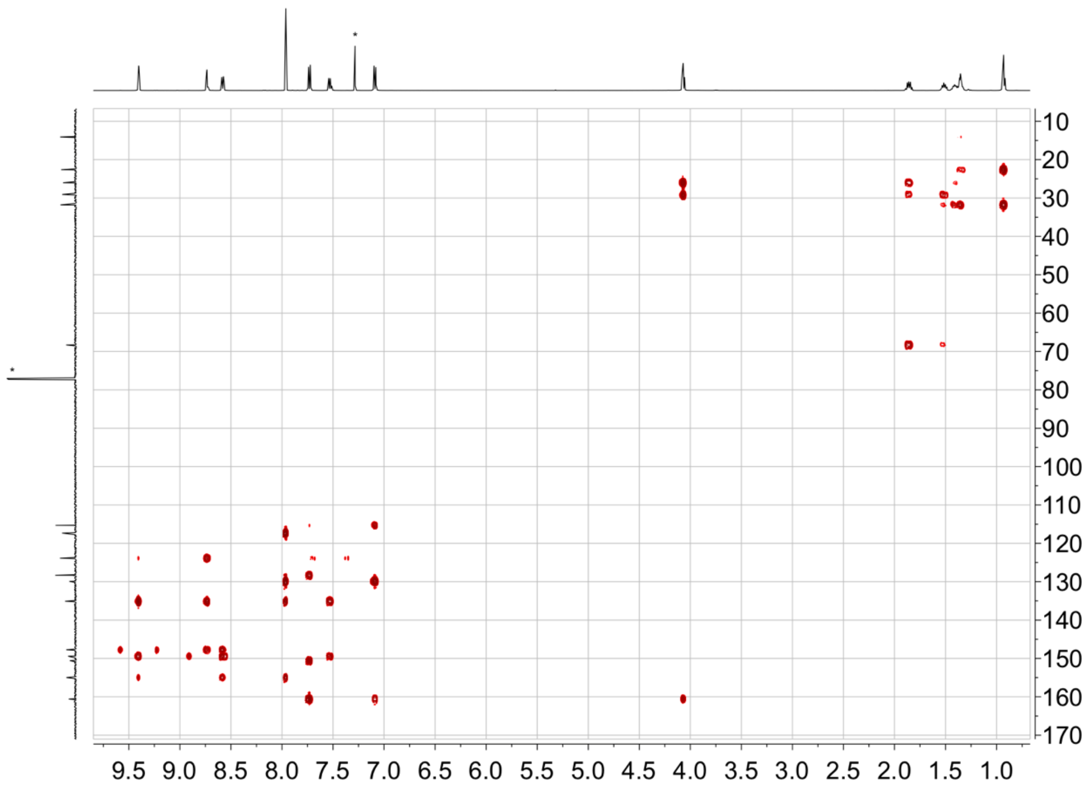

Figure S8. HMBC spectrum of **4** in $CDCl_3$ (500 MHz ^{1}H, 126 MHz ^{13}C{^{1}H}, 298 K). * = $CHCl_3$/$CDCl_3$.

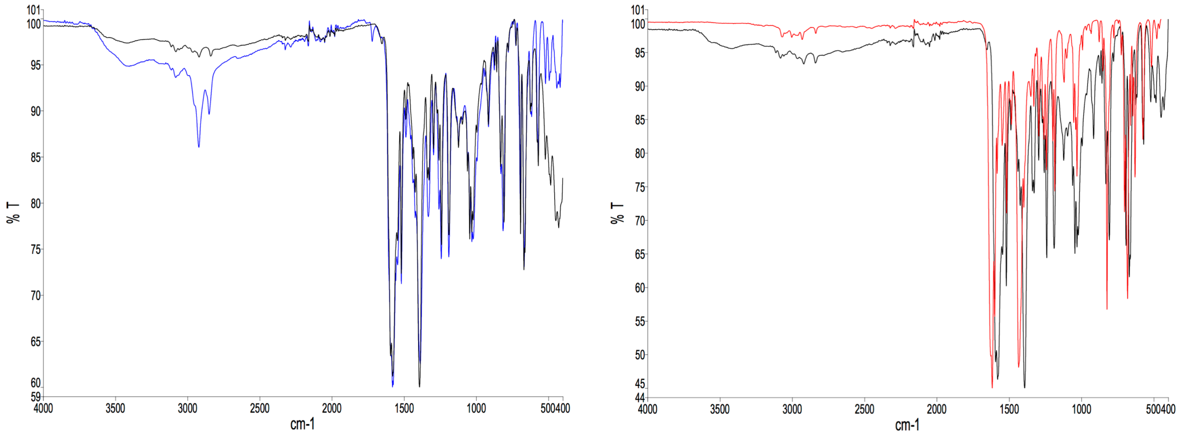

(a) (b)

Figure S9. Normalized FT-IR spectra (solid-state) of the bulk materials from (a) experiment 1 (black line) and experiment 2 (blue line), and (b) experiment 1 (black line, $[\{Cu_4(\mu_3\text{-}OH)_2(\mu\text{-}OAc)_2(\mu_3\text{-}OAc)_2(AcO\text{-}\kappa O)_2(\mathbf{1})_2\}{\cdot}2MeOH]_n$) and the preparative scale reaction (red line, $[Cu_2(\mu\text{-}OAc)_4(\mathbf{1})]_n$).

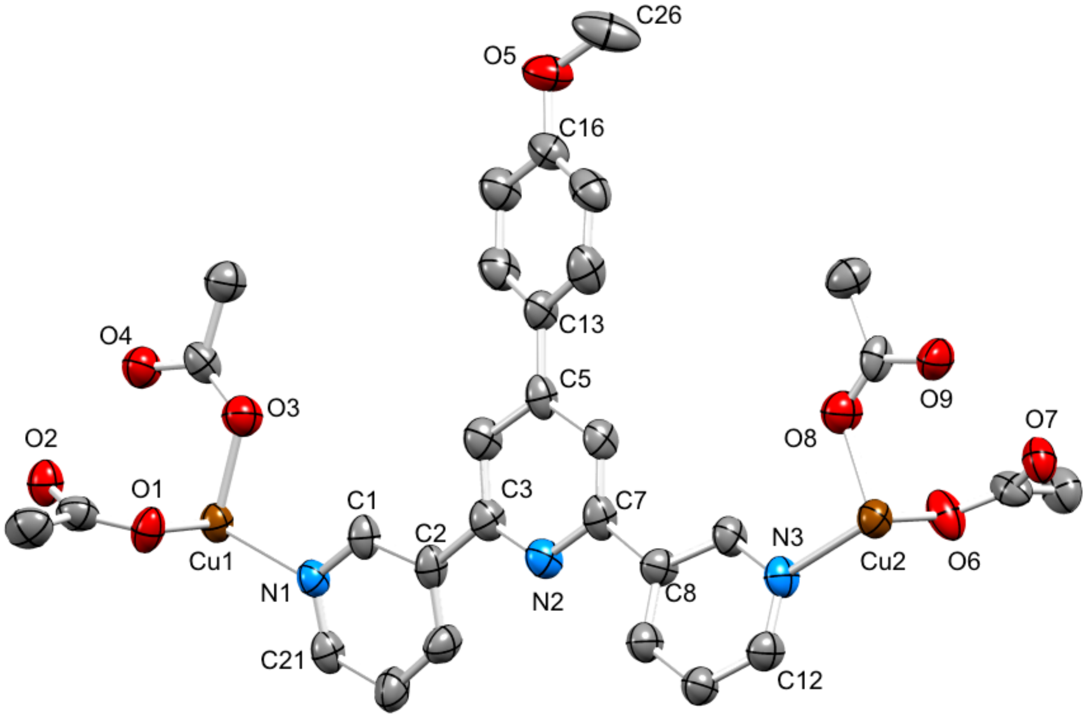

Figure S10. ORTEP-stype representation of the asymmetric unit in $[Cu_2(\mu\text{-}OAc)_4(\mathbf{1})]_n$ with ellipsoids plotted at 40% probability level and H atoms omitted.

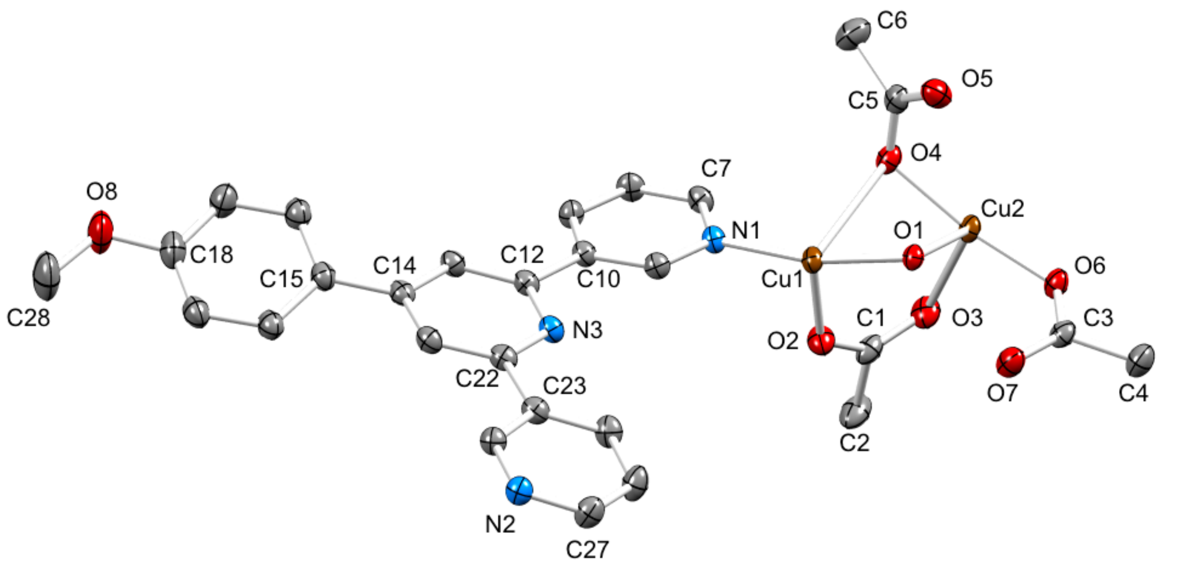

Figure S11. ORTEP-stype representation of the asymmetric unit in {[Cu$_4$(μ_3-OH)$_2$(μ-OAc)$_2$(μ_3-OAc)$_2$(AcO-κ*O*)$_2$(**1**)$_2$]·2MeOH}$_n$ with ellipsoids plotted at 40% probability level and H atoms and solvent molecules omitted.

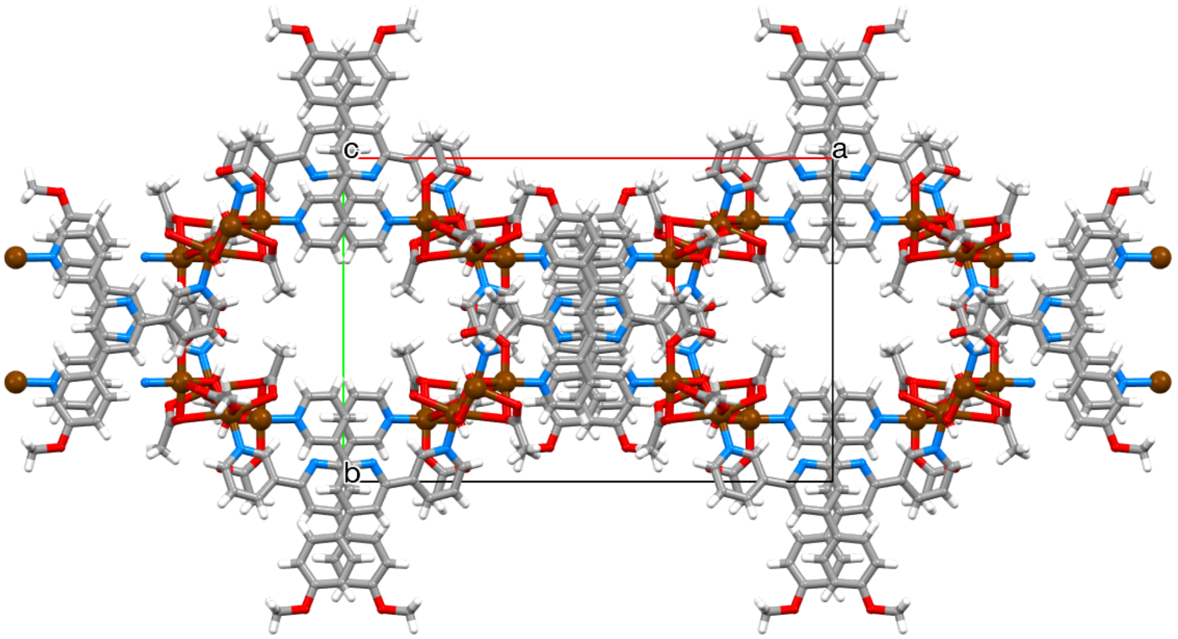

Figure S12 Packing of chains in {[Cu$_4$(μ_3-OH)$_2$(μ-OAc)$_2$(μ_3-OAc)$_2$(AcO-κ*O*)$_2$(**1**)$_2$]·2MeOH}$_n$.

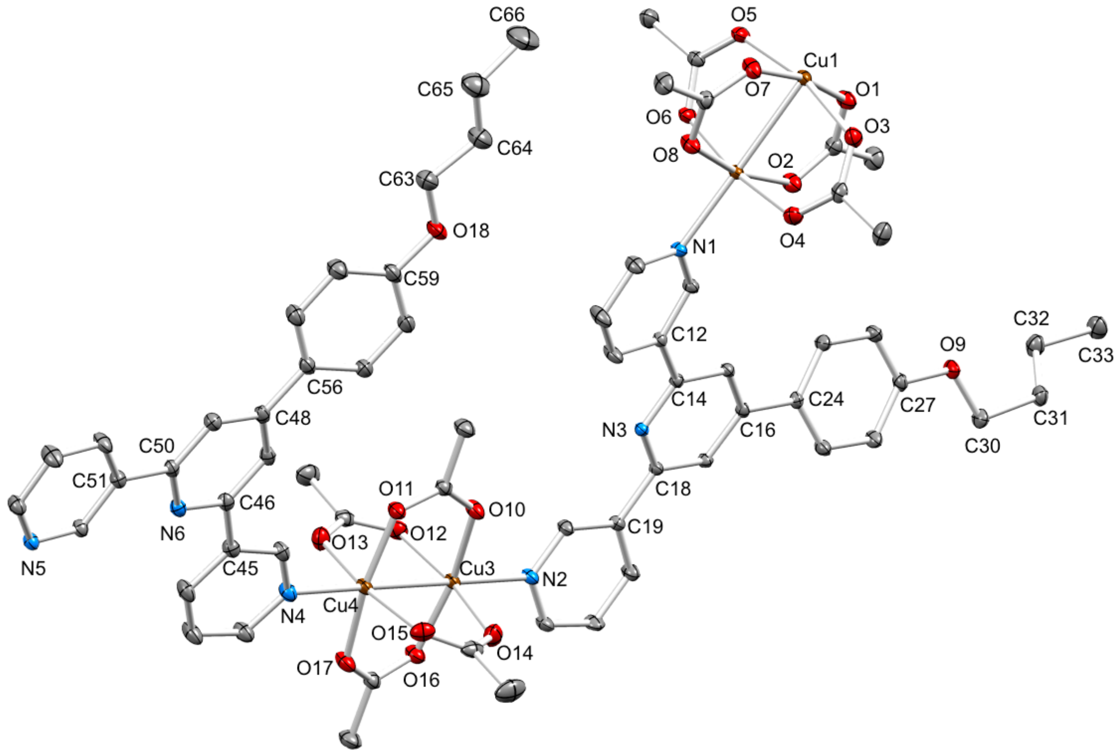

Figure S13. ORTEP-stype representation of the asymmetric unit in $[2\{Cu_2(\mu\text{-}OAc)_4(\mathbf{2})\}\cdot 1.25MeOH]_n$ with ellipsoids plotted at 40% probability level and H atoms and solvent molecules omitted.

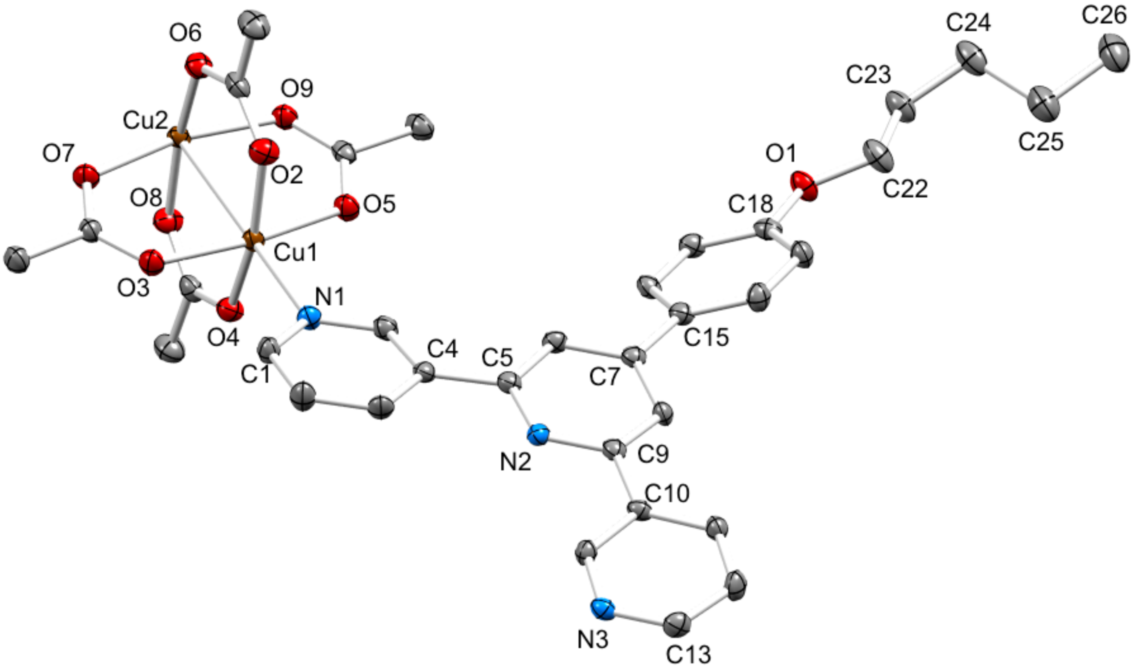

Figure S14. ORTEP-stype representation of the asymmetric unit in $[Cu_2(\mu\text{-}OAc)_4(\mathbf{3})]_n$ with ellipsoids plotted at 40% probability level and H atoms omitted.

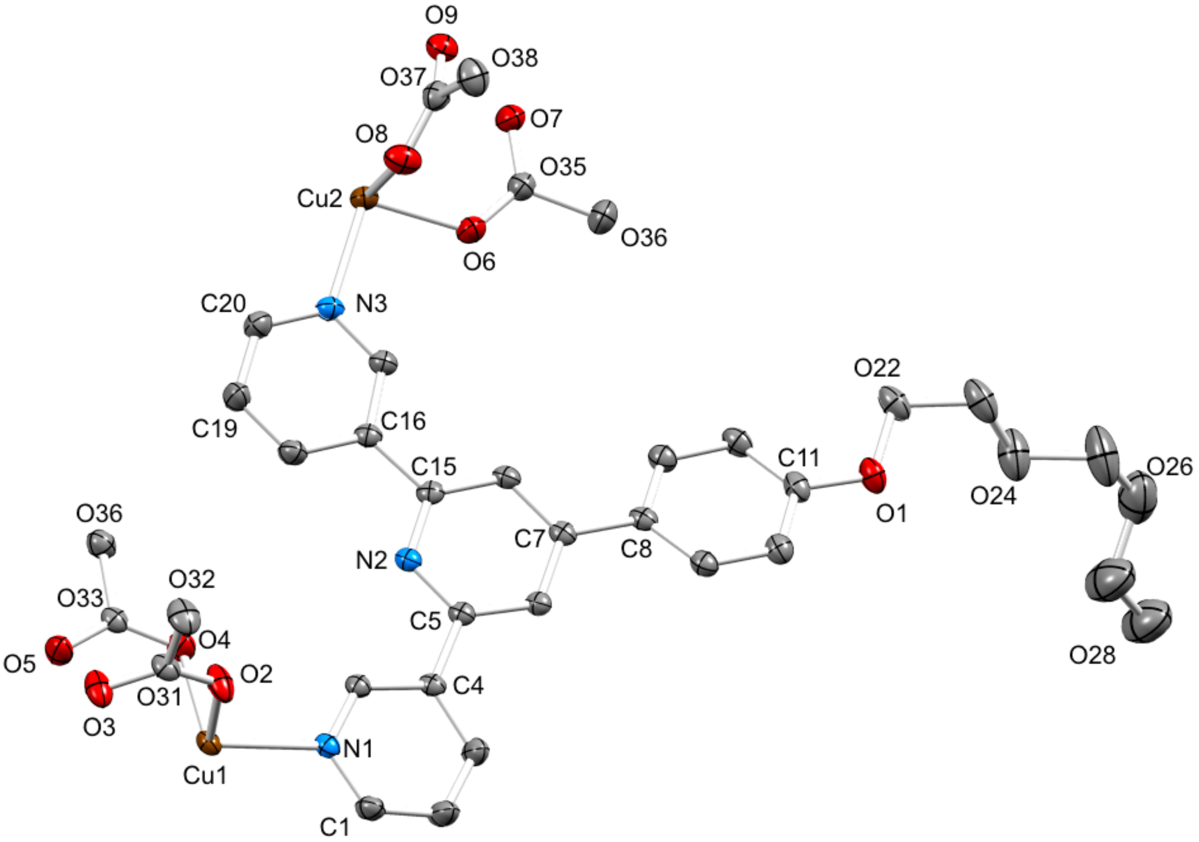

Figure S15. ORTEP-stype representation of the asymmetric unit in [Cu_2(μ-OAc)$_4$(**4**)}·0.2$CHCl_3$]$_n$ with ellipsoids plotted at 40% probability level and H atoms and solvent molecules omitted.

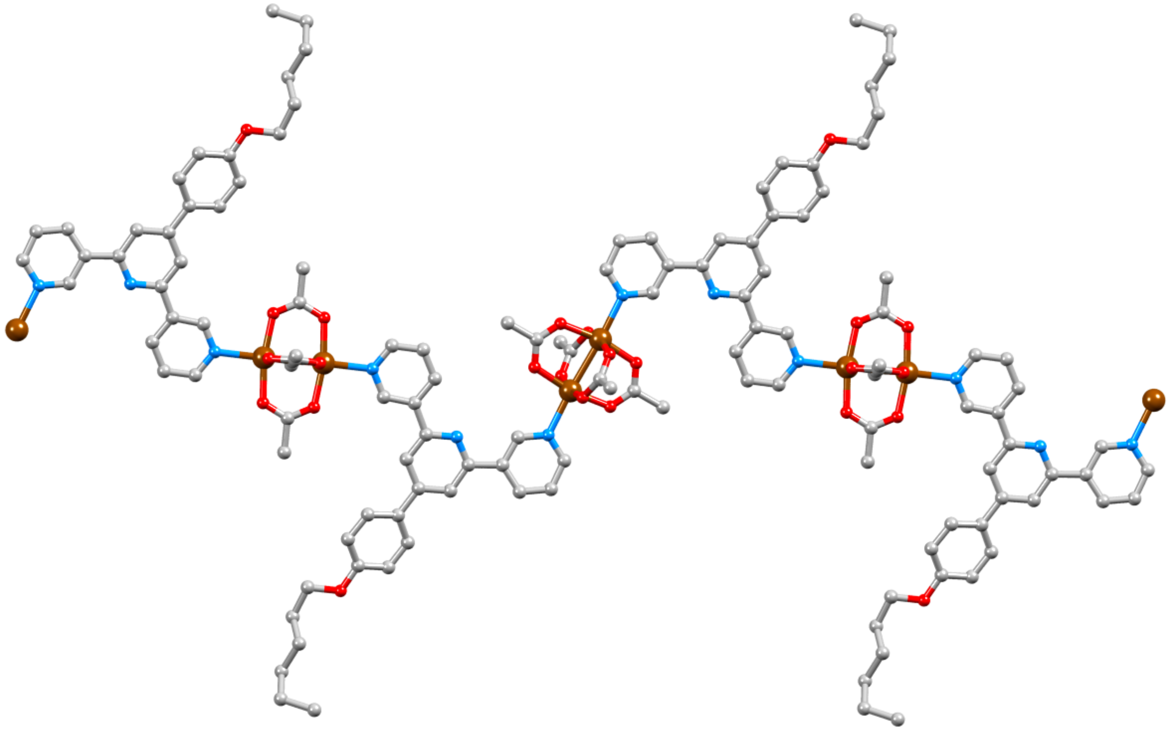

Figure S16. Part of one chain in [Cu_2(μ-OAc)$_4$(**5**)}·0.2$CHCl_3$]$_n$, drawn using data retrieved from the CSD, refcode SADBIL [1].

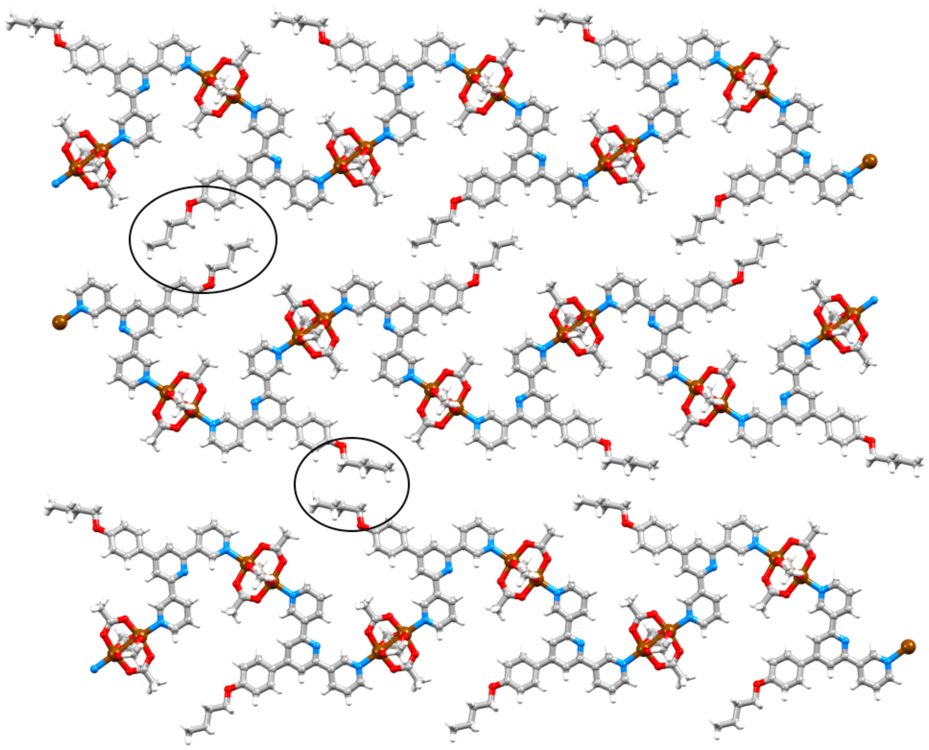

Figure S17. Packing of three adjacent chains (each runs left to right) shown in ball-and-stick representation in $[2\{Cu_2(\mu\text{-}OAc)_4(\mathbf{2})\}\cdot1.25MeOH]_n$. The two independent alkyl...alkyl van der Waals packing interactions are highlighted.

References

1. Li, L.; Zhang, Y.Z.; Yang, C.; Liu, E.; Golen, J.A.; Zhang, G. One-dimensional copper(II) coordination polymers built on 4′-substituted 4,2′:6′,4″- and 3,2′:6′,3″-terpyridines: Syntheses, structures and catalytic properties. *Polyhedron* **2016**, *105*, 115–122. DOI: 10.1016/j.poly.2015.12.042
